# Monitoring Acute Pain in Donkeys with the Equine Utrecht University Scale for Donkeys Composite Pain Assessment (EQUUS-DONKEY-COMPASS) and the Equine Utrecht University Scale for Donkey Facial Assessment of Pain (EQUUS-DONKEY-FAP)

**DOI:** 10.3390/ani10020354

**Published:** 2020-02-22

**Authors:** Machteld C. van Dierendonck, Faith A. Burden, Karen Rickards, Johannes P.A.M. van Loon

**Affiliations:** 1 Department of Equine Sciences, Faculty of Veterinary Medicine, Utrecht University, Yalelaan 114, 3584 CM Utrecht, The Netherlands; J.P.A.M.vanLoon@uu.nl; 2 Department of Nutrition, Genetics and Ethology, Veterinary Faculty, Ghent University, Heidestraat 19, 9820 Merelbeke-Ghent, Belgium; 3 Department of Veterinary Sciences, Faculty of Pharmaceutical, Biomedical and Veterinary Sciences, Antwerp University, Universiteitsplein 1, 2610 Wilrijk-Antwerp, Belgium; 4 The Donkey Sanctuary, Sidmouth, Devon EX10 0NU, UK; faith.burden@thedonkeysanctuary.org.uk (F.A.B.); karen.rickards@thedonkeysanctuary.org.uk (K.R.)

**Keywords:** donkey, acute, pain, assessment, facial expression, composite pain scale

## Abstract

**Simple Summary:**

Pain can be difficult to objectively assess in animals, especially in prey animals that hide their symptoms from others. Donkeys are also known to mask and hide these signs. Owners often describe donkeys as “stubborn”, but veterinarians and equine ethologists describe donkeys more as “stoic”. Among veterinarians it is known that donkeys hide their pain symptoms even more than horses. Therefore, objective and valid pain measurement tools are needed to assess pain in donkeys. In this study, two pain scales (with behavioural elements, physiological elements, responses to interactions, and facial expressions) have been developed and tested in 79 donkeys with various types of acute pain (acute lameness, colic, head-related pain, and postoperative pain) and in 185 healthy pain-free control donkeys. The authors found that the Equine Utrecht University Scale for Donkey Composite Pain Assessment (EQUUS-DONKEY-COMPASS) and the Equine Utrecht University Scale for Donkey Facial Assessment of Pain (EQUUS-DONKEY-FAP) can both be effective to objectively assess various types of acute pain in donkeys and could potentially be used to monitor pain and improve welfare in donkeys.

**Abstract:**

Objective pain assessment in donkeys is of vital importance for improving welfare in a species that is considered stoic. This study presents the construction and testing of two pain scales, the Equine Utrecht University Scale for Donkey Composite Pain Assessment (EQUUS-DONKEY-COMPASS) and the Equine Utrecht University Scale for Donkey Facial Assessment of Pain (EQUUS-DONKEY-FAP), in donkeys with acute pain. A cohort follow-up study using 264 adult donkeys (*n* = 12 acute colic, *n* = 25 acute orthopaedic pain, *n* = 18 acute head-related pain, *n* = 24 postoperative pain, and *n* = 185 controls) was performed. Both pain scales showed differences between donkeys with different types of pain and their control animals (*p* < 0.001). The EQUUS-DONKEY-COMPASS and EQUUS-DONKEY-FAP showed high inter-observer reliability (Cronbach’s alpha = 0.97 and 0.94, respectively, both *p* < 0.001). Sensitivity of the EQUUS-DONKEY-COMPASS was good for colic and orthopaedic pain (83% and 88%, respectively), but poor for head-related and postoperative pain (17% and 21%, respectively). Sensitivity of the EQUUS-DONKEY-FAP was good for colic and head-related pain (75% and 78%, respectively), but moderate for orthopaedic and postoperative pain (40% and 50%, respectively). Specificity was good for all types of pain with both scales (91%–99%). Different types of acute pain in donkeys can be validly assessed by either a composite or a facial expression-based pain scale.

## 1. Introduction

The importance of objective pain assessment in horses and donkeys was reported by Asley, et al. (2005) [1] and Robertson (2006) [2] over a decade ago. Since that date, numerous scientific studies have appeared focusing on (acute) pain assessment in horses. Several reviews [3,4,5] have recently appeared, providing an overview of all the studies on equine pain scoring. However, to date, no studies have described the construction and validation of pain scales for donkeys. This is a major challenge in clinical practice, as in many situations donkeys do not express such obvious pain signs as horses. Reliable and objective pain assessment in donkeys, especially in the field, would be a major step forward enabling early intervention and effective monitoring of response, thereby improving donkey welfare.

The importance of behavioural parameters to assess welfare and pain in working donkeys and mules was described by Pritchard et al. [6]. They described health and behavioural parameters in almost 5000 working horses, donkeys, and mules in developing countries. Regan et al. [7] described the behavioural repertoire of working donkeys as well, with the intention of building an evidence-based ethogram to assess pain in working donkeys. In a recent study [8], these authors assessed behavioural differences in response to analgesic treatment in working donkeys.

Although it is known that donkeys have certain specific variations that make them different to horses, for example their behaviour, physiology, and susceptibility to disease [9], they have many similarities as well. Therefore, it seems logical that the methods and the scales themselves, which successfully designed and validated pain scales for horses, could be used as a starting point for the development of specific pain scales that could be useful and valid for donkeys. Composite pain scales have been described for several types of acute pain in horses (Composite Pain Scale (CPS [10]; Post Abdominal Surgery Pain Assessment Scale (PASPAS) [11]; Equine Utrecht University Scale for Composite Pain Assessment (EQUUS-COMPASS) [12]). In accordance with the development in other species, facial expression-based pain scales for acute pain have been designed for horses as well (the Horse Grimace Scale [13]; the Equine Pain Face [14]; and the Equine Utrecht University Scale for Facial Assessment of Pain [12]) to assess various types of acute pain). Although pain assessment in donkeys has been studied by several authors [1,6,7,15], no attempts have been made to develop and validate structured pain scales for this species. Since a donkey’s primary anti-predatory behaviour is freezing, they mask many signs of pain [9], meaning that horse scales may need to be adapted to pick-up subtle signs of pain in the donkeys. 

The aim of the current study was to develop a composite pain scale for assessment of acute pain in donkeys, the Equine Utrecht University Scale for Donkey Composite Pain Assessment (EQUUS-DONKEY-COMPASS), and a facial expression-based pain scale, the Equine Utrecht University Scale for Donkey Facial Assessment of Pain (EQUUS-DONKEY-FAP). The equine EQUUS-COMPASS and EQUUS_FAP have proved reliable in scientific studies and during practical applications. Therefore, they were combined with the earlier mentioned donkey-specific studies, interviews, and a pilot project as a starting point for the development of the donkey pain assessment scales. As previous work in horses had identified that the sensitivity of different types of pain scales was dependent on the type of acute pain experienced [5], two different pain scales were developed and evaluated as part of this study using donkeys with various types of acute pain and healthy control animals at two different professional Donkey Rescue locations.

## 2. Materials and Methods

### 2.1. Subsection

The institutional Ethics Committee on the Care and Use of Experimental Animals approved the study design in compliance with Dutch and English legislation on animal experimentation. Because the procedures used in this study only consist of behavioural observations and physiologic assessments (heart rate, breathing rate, borborygmi, rectal temperature) that are routinely taken in the clinical setting and are deemed not likely to cause pain, suffering, or distress or lasting harm equivalent to, or higher than, that caused by the introduction of a needle (article 1.5f EU directive 2010/63/EU), ethical approval was granted without a formal application and hence no official approval number was given. Written owner’s consent was obtained for all animals participating in this study. 

### 2.2. Animals 

Seventy-nine donkeys presenting with acute pain (acute colic, acute orthopaedic, or acute head-related pain and animals directly after surgery for postoperative pain) that were observed between 2016 and 2018 were enrolled in this study (Table 1). Patients were assessed by a veterinarian and only after approval and diagnosis enrolled in the study. Only donkeys with a new episode of colic (*n* = 12) (impaction colic or other colic) were enrolled. Orthopaedic cases (*n* = 25) comprised mainly donkeys with acute laminitis and solar abscesses; the acute head-related injuries/pain (*n* = 18) cases comprised donkeys with acute uveitis and corneal injuries/ulcers and dental pain, and donkeys in the group of postoperative pain (*n* = 24) comprised mainly those who had undergone sarcoid removal, castration (*n* = 7), and dental extractions (both under general anaesthesia or under sedation and local blocks). More details of the patients can be found in Supplementary Materials Table S1. Where possible, new patients were assessed before analgesic treatment, which was provided by the treating veterinarian (not known to the observers) (mainly consisting of Non-steroidal Anti-inflammatory Drugs (NSAIDs) in all patients, combined with local anaesthetics and opioids in surgical patients). For all donkeys the time of the pain assessment and the time and dose of the medication provision was known. The donkeys were housed at The Donkey Sanctuary in the United Kingdom (76 patients) or at the donkey shelter “Anegria” in Belgium (3 adult >7-year-old) castration patients). Many donkeys have a (very) strong attachment to usually one specific close companion [9]; therefore, in principle for each patient donkey at least two control donkeys, that were deemed free from pain after clinical examination by a veterinarian, were assigned: the close companion (“partner control”) and a matched control (matching as far as possible) from the same herd (“herd control”). More details of the controls can be found in Supplementary Materials Table S2. In the majority of cases the control donkeys were assessed straight after the patient’s assessment. If this was not possible (due to darkness or other technical reasons) the control donkey(s) were assessed at the earliest possible time comparable with the time of the patient’s assessment. In total, 185 healthy (179 in the United Kingdom, 6 in Belgium), pain-free control donkeys from the same location were included (Table 1). 

### 2.3. The Equine Utrecht University Scale for Donkey Composite Pain Assessment Facial Assessment of Pain (EQUUS-DONKEY-COMPASS) 

The EQUUS-DONKEY-COMPASS is based on the EQUUS-COMPASS that has been described for the assessment of horses with acute colic pain [12,16]. Since the aim was to construct a donkey scale not only for acute colic, elements of the equine COMPASS scale were supplemented with parameters related to lameness, based on an equine acute orthopaedic pain scale [10]. In addition, relevant parameters for donkeys were identified using a donkey-specific ethogram based on the literature [1,7,9,17] and the expert opinion of donkey specialists (veterinarians predominantly working with donkeys in the Netherlands and abroad were included). All elements were systematically described and tested in a pilot project (not published). The EQUUS-DONKEY-COMPASS is a multifactorial simple descriptive scale based on 20 parameters scored by direct observation for 5 min. It includes physiologic parameters, responses to stimuli, and spontaneous behavioural parameters (Table 2). 

### 2.4. The Equine Utrecht University Scale for Donkey Facial Assessment of Pain (EQUUS-DONKEY-FAP)

**Figure 1:**
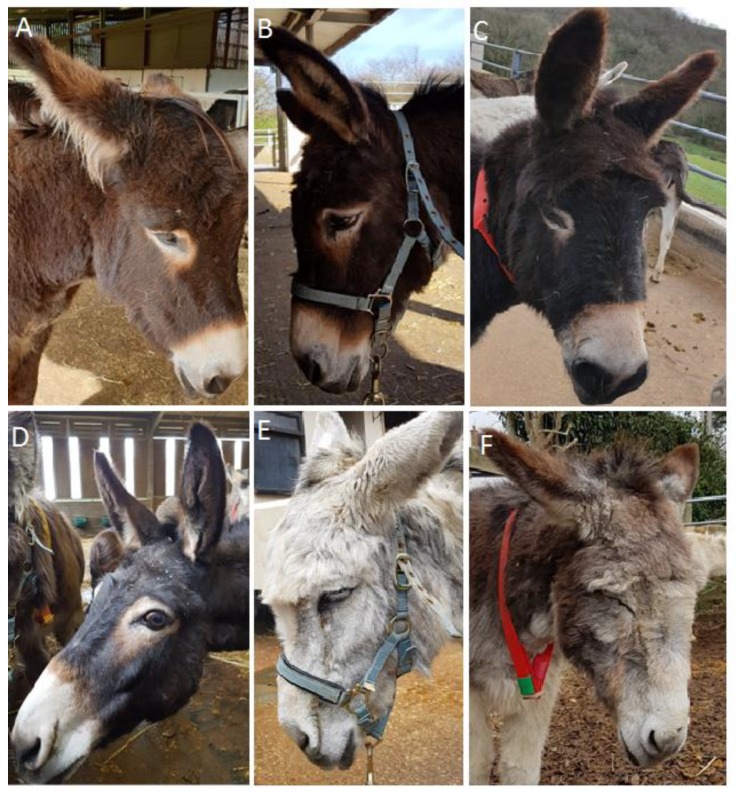
Examples of facial characteristics of donkeys with acute pain: backwards-directed ears (**A**), more open eyelids (**B**), obviously opened nostrils (**C**), obviously more opened eyes with sclera visible (**D**), obviously lifted corners of the mouth (**E**), obvious orbital tightening of eyelids (**F**).

The EQUUS-DONKEY-FAP is based on the EQUUS-FAP, that has been designed and validated for the assessment of horses with acute colic [12,16] and has been described in horses with acute head-related pain [18], and in horses with acute orthopaedic or trauma pain and with postoperative orthopaedic pain [19]. Several additional parameters were included in the equine FAP scale after a pilot project, which identified differences in behavioural pain expression between horses and donkeys. The EQUUS-DONKEY-FAP is a multifactorial simple descriptive scale based on 12 parameters scored by direct observation for 2 min (Table 3). Figure 1 shows examples of donkeys with facial expression characteristics that can be seen in donkeys with acute pain. 

### 2.5. Pain Observations

Before starting the study, the observers were given training in the use of both pain scales in donkeys without signs of pain (subjects not subsequently included in the study) under the supervision of two of the authors (M.v.D. and T.v.L.). Basically, the same protocol as used in the previous equine studies [12,16] was followed. Observations were performed by a total of three groups of two observers (three periods: in each of the periods two veterinary master students were involved in the observations together). The duo of students scored simultaneously but independently of each other. To avoid initial freezing of the donkeys and/or disturbance due to just the presence of unknown human observers, both the patient and control donkeys were first observed from a comfortable distance (3–5 m when possible). Only when the donkey was neither freezing (anymore) nor sleeping, the observations started for 5 min (EQUUS-DONKEY-COMPASS) followed by 2 min (EQUUS-DONKEY-FAP)—still from the same distance. During this time the frequencies of those elements which required counting (see Table 2 and Table 3) or scores for elements with lowest possible human influence (like respiratory rate, focus or posture etc.) were determined. After these observations the donkey was approached at a slow pace and in a quiet manner, in order to be able to determine the reactivity responses (see Table 2 and Table 3) and the remaining clinical parameters (see Table 2). Donkeys were observed in their home environment or, for the surgical patients, in a stable in the veterinary clinic where they were housed together with a close companion. Due to the field conditions it was impossible for the observers to be blinded to either the status of the donkey (patient or control) or its medical condition. For the EQUUS-DONKEY-COMPASS score it will never be possible to be blinded, especially for mild or severe trauma, colic, or orthopaedic patients. After the initial pain assessment, wherever possible, the animals were followed for 3 days (if not discharged earlier), with pain scoring in the morning and afternoon. The observers were not involved in day-to-day patient care and were unaware of the analgesic treatment protocol at the moment of pain assessment. The surgical patients were assessed before surgery for baseline assessments. The post-operative assessments were 4 and 8 h after the end of the surgery, after this day, and in line with the other types of pain, if possible (not discharged earlier) they were followed for 3 days. 

### 2.6. Data Processing and Statistical Analysis

All pain scoring data are expressed as medians and quartiles. Differences in ages between patients and control animals were statistically tested for normality by the Shapiro Wilks test. Since the age was not normally distributed in some subgroups, the distribution of age within all subgroups was tested by means of Mann–Whitney *U* tests: none of the patients–control comparisons differed from each other. The possible confounding correlation between age and a pain score was analysed by means of Spearman’s rho (See Supplementary Materials Table S3). All scores of patients and controls can be found in the supplementary material Tables S4–S7 (Table S4: patients EQUUS-DONKEY-COMPASS scores; Table S5: control donkey EQUUS-DONKEY-COMPASS scores; Table S6: patients EQUUS-DONKEY-FAP scores; Table S7: control donkey EQUUS-DONKEY-FAP scores). Differences in pain scores between partner control and herd control animals were tested using Mann–Whitney *U* tests. As these control groups were not different from each other, they were pooled and differences between the pooled control animals and patients were analysed using the Mann–Whitney *U* tests as well. For colic pain, acute orthopaedic pain, and facial pain, the most painful condition—which was at the initial presentation—was used (T0a), while for postoperative pain the score at T0b (= 4 h after surgery) was used, when it was deemed to be most painful. For the four subgroups of patients, pain scores of patients and pooled control animals were analysed using Mann–Whitney *U* tests. Data of castration patients (as part of the group of postoperative pain) were analysed separately and compared to control animals. Cut-off values for both pain scales between patients and control animals were determined based on pilot data and were set at ≥5 for EQUUS-DONKEY-COMPASS and ≥2 for EQUUS-DONKEY-FAP to obtain maximal differentiation between donkeys with acute pain and healthy animals. Sensitivity (the proportion of animals with acute pain with a pain score equal or higher than the cut-off value), specificity (the proportion of non-pain affected animals correctly identified as not in pain), and positive and negative predictive values (respectively the probability that pain was indeed present when the test was positive or the probability that pain was indeed not present when the test was negative) were determined using these cut-off values. Weighting factors for individual parameters of both scales were determined using cut-off values of equal or higher than 1 for individual parameters, with weighting factor = 0 if sensitivity or specificity were <25%, weighting factor = 1 if sensitivity and specificity were between 25% and 50%; weighting factor = 2 if sensitivity and specificity were between 50% and 75%; and weighting factor = 3 if both sensitivity and specificity were ≥75%. Effects over time for both pain scores were only displayed visually and not statistically analysed due to differences in group sizes. Statistical analysis was performed using commercially available software (SPSS version 20.0, IBM). Statistical significance was accepted at *p* < 0.05. 

## 3. Results

### 3.1. Inter-observer Reliability 

**Figure 2:**
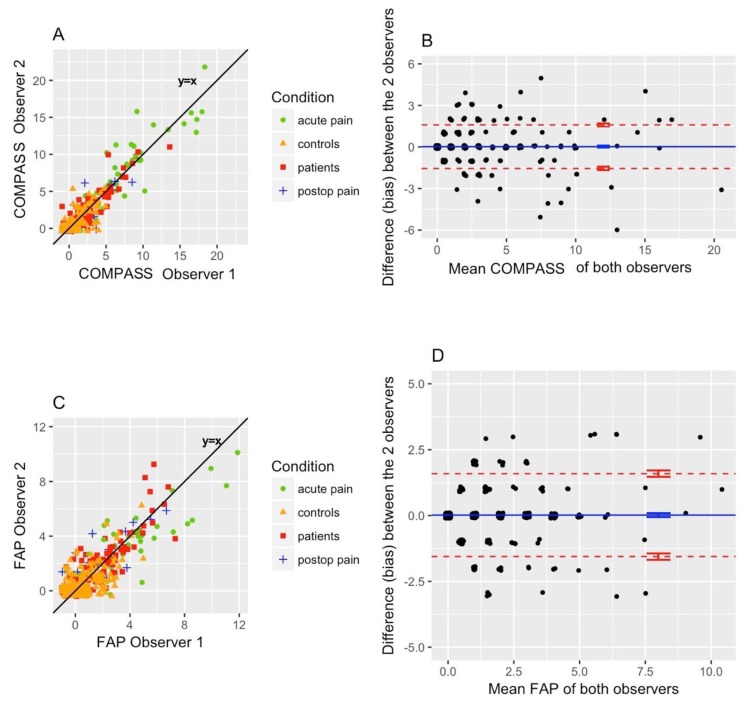
Inter-observer reliability. Equine Utrecht University Scale for Donkey Composite Pain Assessment (EQUUS-DONKEY-COMPASS): Cronbach’s alpha = 0.97 (*p* < 0.001), bias = 0.02, and limits of agreement of −1.9 to +2.0 (*n* = 497) (**A**,**B**). Equine Utrecht University Scale for Donkey Facial Assessment of Pain (EQUUS-DONKEY-FAP): Cronbach’s alpha = 0.94 (*p* < 0.001), bias = 0.02, and limits of agreement of −1.55 to +1.59 (*n* = 497) (**C**,**D**).

Figure 2 shows the results of correlation analysis between the different pain scores of two independent observers. Both pain scales showed a strong and significant correlation (Cronbach´s alpha = 0.97, *p* < 0.001 for EQUUS-DONKEY-COMPASS, Cronbach´s alpha = 0.94, *p* < 0.001 for EQUUS-DONKEY-FAP). 

### 3.2. Relation of Age with Pain Scores

There was no confounding relationship between EQUUS-DONKEY-COMPASS and EQUUS-DONKEY-FAP scores and age within the different pain groups. The Spearman’s rho’s for both pain scales within the different patient pain groups ranged between –0.36 and 0.27; their *p* values ranged between 0.088 and 0.939 see (Supplementary Materials Table S3).

### 3.3. Differences between Subgroups of Control Animals and Patients

**Figure 3:**
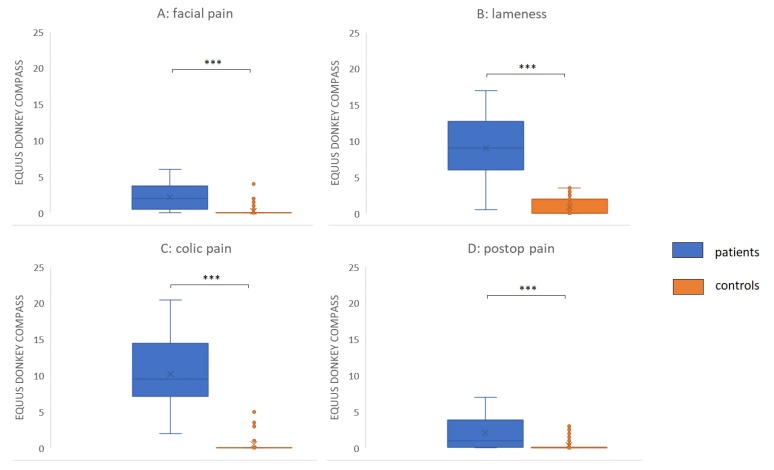
EQUUS DONKEY COMPASS patients versus controls. **(A**) Facial pain (*n* = 18 patients versus = 39 controls), (**B**) Orthopaedic pain (*n* = 25 patients versus *n* = 68 controls); (**C**) Colic pain (*n* = 12 patients versus *n* = 28 controls), (**D**) Postoperative pain (*n* = 24 patients versus *n* = 50 controls). Lines in boxes show median scores; x—mean value; boxes show 25–75th percentiles; error bars show 5–95th percentiles. *** *p* < 0.001.

**Figure 4:**
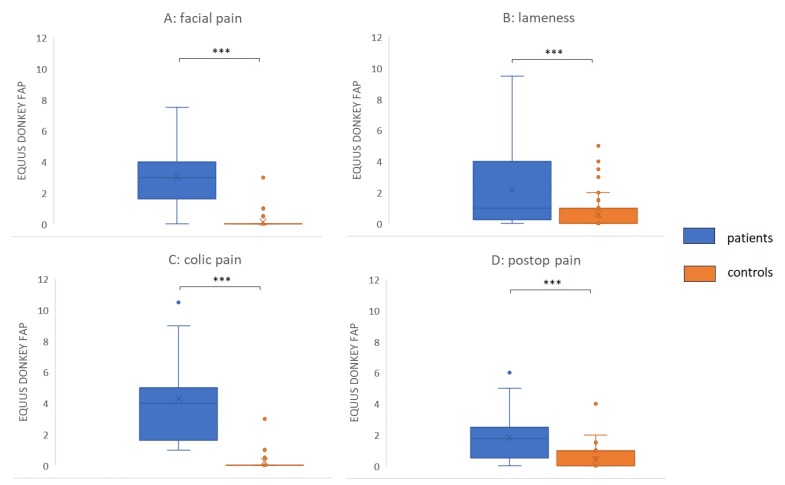
EQUUS-DONKEY-FAP patients versus controls. **(A**) Facial pain (*n* = 18 patients versus *n* = 39 controls), (**B**) Orthopaedic pain (*n* = 25 patients versus *n* = 68 controls); (**C**) Colic pain (*n* = 12 patients versus *n* = 28 controls), (**D**) Postoperative pain (*n* = 24 patients versus *n* = 50 controls). Lines in boxes show median scores; x—mean value; boxes show 25th–75th percentiles; error bars show 5th–95th percentiles. ***—*p* < 0.001.

**Figure 5:**
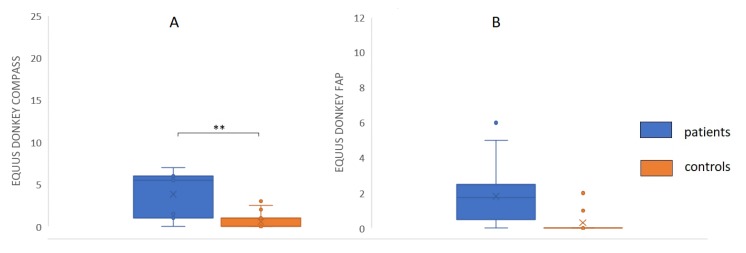
EQUUS-DONKEY-COMPASS) and EQUUS-DONKEY-FAP after surgical castration. (**A**) EQUUS DONKEY COMPASS, (**B**) EQUUS-DONKEY-FAP (*n* = 7 patients versus *n* = 16 controls). Lines in boxes show median scores; boxes show 25th–75th percentiles; error bars show 5th–95th percentiles. * *p* < 0.05.

Both EQUUS-DONKEY-COMPASS and EQUUS-DONKEY-FAP scores showed significant differences between control animals and patients when comparing all animals (*p* < 0.001, *n* = 264). All four subgroups of patients showed higher pain scores compared to their control animals for EQUUS-DONKEY-COMPASS and for EQUUS-DONKEY-FAP) (*p* < 0.001 for all subgroups) (Figure 3 and Figure 4). When the castration patients and their controls were analysed separately, donkeys showed significantly higher COMPASS and FAP scores 4 h after surgical castration (*n* patients = 7, *n* controls = 16; *p* = 0.01, *p* < 0.05, respectively) compared to their controls at that time (Figure 5). 

### 3.4. Effects Over Time in Patient Groups.

**Figure 6:**
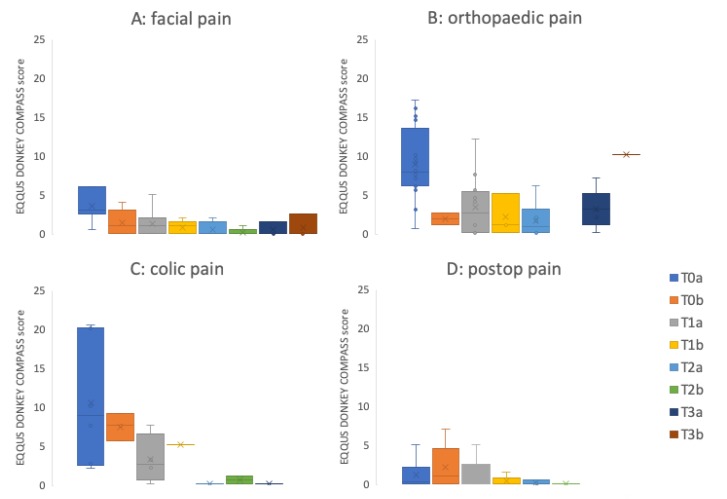
EQUUS-DONKEY-COMPASS mean patient scores over time. (**A**) Facial pain (*n* patients on Day 0: T0a = 7, T01b = 5; Day 1: T1a = 15, T1b = 11; Day 2: T2a = 5, T2b = 5; Day 3: T3a = 3, T3b = 3), (**B**) Orthopaedic pain (*n* patients on Day 0: T0a = 16, T01b = 2; Day 1; T1a = 12, T1b = 3; Day 2: T2a = 6, T2b = 0; Day 3: T3a = 5, T3b = 1); (**C**) Colic pain (*n* patients on Day 0: T0a = 6, T01b = 3; Day 1; T1a = 4, T1b = 2; Day 2: T2a = 2, T2b = 2; Day 3: T3a = 1, T3b = 0), (**D**) Postoperative pain (n patients before surgery: T0a = 22; *n* patients post-surgery: T0b = 23; Day 1; T1a = 17, T1b = 5; Day 2: T2a = 3, T2b = 1; Day 3: T3a = 0, T3b = 0). The ‘a’ score was determined in the morning; the ‘b’ score was determined in the afternoon. Lines in boxes show median scores; x = mean value; boxes show 25th–75th percentiles; error bars show 5th–95th percentiles. T0a: admission to clinic for facial pain, orthopaedic- and colic pain patients, baseline assessment before surgery for surgery patients, T0b: afternoon of first day after admission to clinic for facial pain, orthopaedic- and colic pain patients, first assessment 4 hours after surgery for surgical patients. T1a: morning assessment of day 1, T1b: afternoon assessment of day 1. T2a: morning assessment of day 2, T2b: afternoon assessment of day 2. T3a: morning assessment of day 3, T3b: afternoon assessment of day 3.

**Figure 7:**
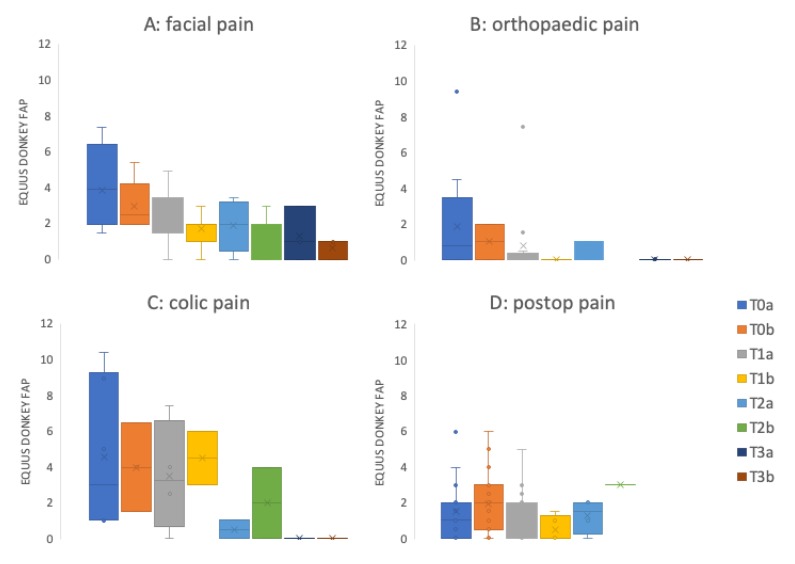
EQUUS-DONKEY-FAP mean patient scores over time. (**A**) Facial pain (*n* patients on Day 0: T0a = 7, T01b = 5; Day 1: T1a = 15, T1b = 11; Day 2: T2a = 5, T2b = 5; Day 3: T3a = 3, T3b = 3), (**B**) Orthopaedic pain (*n* patients on Day 0: T0a = 16, T01b = 2; Day 1; T1a = 12, T1b = 3; Day 2: T2a = 6, T2b = 0; Day 3: T3a = 5, T3b = 1); (**C**) Colic pain (*n* patients on Day 0: T0a = 6, T01b = 3; Day 1; T1a = 4, T1b = 2; Day 2: T2a = 2, T2b = 2; Day 3: T3a = 1, T3b = 0), (**D**) Postoperative pain (patients before surgery: T0a = 22; patients post-surgery: T0b = 23; Day 1; T1a = 17, T1b = 5; Day 2: T2a = 3, T2b = 1; Day 3: T3a = 0, T3b = 0). The ‘a’ score was determined in the morning; the ‘b’ score was determined in the afternoon. Lines in boxes show median scores; x= mean value; boxes show 25th–75th percentiles; error bars show 5th–95th percentiles. T0a: admission to clinic for facial pain, orthopaedic- and colic pain patients, baseline assessment before surgery for surgery patients, T0b: afternoon of first day after admission to clinic for facial pain, orthopaedic- and colic pain patients, first assessment 4 hours after surgery for surgical patients. T1a: morning assessment of day 1, T1b: afternoon assessment of day 1. T2a: morning assessment of day 2, T2b: afternoon assessment of day 2. T3a: morning assessment of day 3, T3b: afternoon assessment of day 3.

Figure 6 and Figure 7 show the mean pain scores over a maximum of 3 days for different groups of patients (facial pain (*n* = 7), orthopaedic pain (*n* = 16), colic pain (*n* = 7), and postoperative pain (*n* = 22 patients before surgery; *n* = 23 post-surgery). 

### 3.5. Sensitivity, Specificity, and Weighting Factors for Individual Parameters

Overall sensitivity of the EQUUS-DONKEY-COMPASS was moderate (50.6%), while overall specificity was high (99.5%, *n* = 79). For the EQUUS-DONKEY-FAP, overall sensitivity was moderate (57.0%) while overall specificity was high as well (90.8%, *n* = 185), with cut-off values of 5 (COMPASS) and 2 (FAP). Sensitivity values for both colic and orthopaedic pain were 83% and 88%, respectively, in the EQUUS-DONKEY-COMPASS but 17% and 21%, respectively, for head-related pain and postoperative pain. For EQUUS-DONKEY-FAP the sensitivity values for colic and head-related pain were 75% and 78%, respectively, while they were 40% and 50%, respectively, for orthopaedic pain and postoperative pain. For castration patients only, sensitivity was 57.0% for EQUUS-DONKEY-COMPASS and 42.9% for EQUUS-DONKEY-FAP (Table 4). Weighting factors for the individual parameters of both scales (based on cut-off values of 1) are shown in Table 5 and Table 6. 

## 4. Discussion

The current study shows that the EQUUS-DONKEY-COMPASS and EQUUS-DONKEY-FAP, a composite pain scale and a facial expression pain scale, respectively, prove to be valid and clinically applicable pain scales for donkeys with different types of acute pain under field conditions. This study was performed to assess the ability of these scales to provide an objective evaluation of the severity of pain symptoms in donkeys with acute somatic or visceral pain represented by orthopaedic, head related, surgical, or colic-related pain. Both scales showed a high inter-observer reliability with clinically acceptable limits of agreement, and could differentiate between healthy control animals and animals with acute pain under field conditions. Care was taken to take time and keep any disturbance by observers to a minimum while assessing the donkeys to make sure that the subtle signals were observed, since the natural freezing reaction was induced very easily. Locomotion could only be objectively assessed by using positive reinforcement as suggested by Burden and Thiemann [9].

Despite the overall significant difference between scores of patients and controls, the overall sensitivity was below 60% for both scales, while the specificity was higher than 89.7% for both scales. The EQUUS–DONKEY-COMPASS scale differentiated with a sensitivity of >83.3% between donkeys with acute orthopaedic pain or colic and their pain-free control animals. Differentiation between patients and controls within the postoperative pain and facial pain subgroups was poor (<21%) for the COMPASS scale. On the other hand, the EQUUS-DONKEY-FAP scale differentiated well between the donkeys with acute head-related pain and donkeys with colic and their controls (sensitivity for both >75%). Our findings imply that acute orthopaedic pain would be best assessed with the EQUUS-DONKEY-COMPASS scale, while donkeys with acute head-related pain would be best assessed with the EQUUS-DONKEY-FAP. This corresponds to the findings in horses, where acute head-related pain is best assessed by means of facial expression-based pain scales as well [18]. For donkeys with acute colic, both pain scales could be reliably used to assess acute visceral pain. This corresponds to earlier findings in horses as well [12,16].

For donkeys with post-operative pain, both scales had difficulties with differentiating patients from control animals. However, different types of surgery with different levels of surgical invasiveness were taken together in this group of animals. Most of the surgeries comprised sarcoid removals (*n* = 10, COMPASS score 0.9 ± 0.7; FAP score 1.6 ± 2.1 four hours after surgery) or castrations (*n* = 7, COMPASS score 3.9 ± 2.9; FAP score 2.4 ± 1.7; four hours after surgery). Additionally, analgesic medication was always provided pre- and post-surgery. These factors could explain why the postoperative patients did not reach high sensitivity in both pain scores. However, when post-castration pain was evaluated separately and compared to their control animals, donkeys did show both increased FAP and COMPASS scores. Both scores were higher compared to control animals, and sensitivity to differentiate between castration pain and control animals increased from 21% to 57% for the COMPASS scores (with a specificity of 100%), while it did decrease somewhat for the FAP scores (sensitivity 50% to 43% with a specificity of 94%). Donkeys in the current study were anaesthetised with protocols including preoperative administration of NSAIDs, general anaesthesia, and intratesticular administration of local anaesthetics. In the study by Dalla Costa et al. [13], stallions were castrated under general anaesthesia with preoperative NSAIDs, but without intratesticular local anaesthetics. These stallions showed increased facial pain scores after surgery. In the study by Abass et al. [20], stallions that were anaesthetised with a protocol including intratesticular local anaesthetics did not show increased pain scores after surgery, nor did they show increased inflammatory markers, while the stallions that did not receive this intratesticular block showed higher pain scores. In another study [21], donkeys showed increased CPS scores (a composite pain scale validated for horses [10] after surgical castration under general anaesthesia, with or without local intratesticular anaesthesia. Although the donkeys in the current study showed increased composite and facial pain expression scores, they only showed an increased sensitivity in the COMPASS scores after surgical castration. This indicates that castration pain in donkeys might best be assessed by means of the EQUUS-DONKEY-COMPASS scale.

Physiological parameters in the EQUUS-DONKEY-COMPASS showed low sensitivity and low specificity, therefore, they received weighting factors of 0 in most types of pain. This means that according to this study, composite pain scales in donkeys could be used to assess acute pain without requiring the assessment of physiological parameters. This corresponds with findings in previous studies in horses with acute colic pain [12] and acute orthopaedic pain [22]. The lack of an added value of assessing physiological parameters in composite pain scales implies that horse and donkey owners, who are not always capable of assessing heart rate and abdominal sounds, can be accurately trained to use these adapted pain scales in their animals under field conditions. Only in donkeys with acute colic pain did the parameter “abdominal sounds” obtain a weighting factor of 3 (because of high sensitivity and specificity). The EQUUS-DONKEY-COMPASS proved useful to assess pain in all donkeys without inclusion of physiological variables.

Facial expressions in donkeys show a lot of similarities with those of horses with acute pain. The Facial Action Coding System (FACS) for horses (EquiFacs) has been described by Wathan et al. [23] and various pain scales that are based on expression of facial characteristics have been investigated [12,13,14]. Although the facial action coding system for donkeys has not been described, donkeys and horses show various similarities in their facial expression. Therefore, the ethogram that has been used in the EQUUS-DONKEY-FAP is very similar to the equivalent in horses. In the EQUUS-DONKEY-FAP, ear position and response to sounds have been split. Startle reflex, headshaking and sweating behind the ears have been added, based on the pilot study. However, these last parameters did not show added value for the different types of pain in donkeys, so following future confirmation studies they could be discarded. The parameters that showed most importance were position of the eyelids, nostrils, corners of the mouth, ear position and focus (this last parameter specifically for colic pain). EQUUS-DONKEY-FAP was the pain scale of choice for donkeys with acute colic pain and facial pain, which shows clear similarities with the use of the related EQUUS-FAP in horses with these types of pain.

Limitations of the current study were the fact that direct and unblinded observations were performed by the observers since this study was performed under field conditions (although the observers were not involved with clinical decision-making about analgesic treatment of patients). To date, this approach has more often been chosen when clinical studies with horses were conducted and pain behaviour was assessed as well. However, the fact that the observers were aware of the condition that the donkey was experiencing could always influence pain scoring to some extent, because of expectation bias [24]. To date, it has not been proven possible to score reliably from photos or videos that are taken from horses experiencing pain (unpublished data). Since in the EQUUS-DONKEY-COMPASS locomotion and postural assessments are included, so by definition it is difficult to score blinded, the same holds when trauma is involved. In the future, this should be counteracted if pain scoring from videos that are randomized and blinded for the observers should become feasible or automated facial recognition techniques can successfully be applied in donkeys and horses as suggested by McLennan [25]. Future studies will be directed towards assessment of chronic pain in horses and donkeys. Because chronic pain may be more subtle and nonspecific, this may prove to be an even more challenging task compared to assessment of acute pain.

## 5. Conclusions

The EQUUS-DONKEY-COMPASS and EQUUS-DONKEY-FAP are composite and facial expression-based pain scales that provide valid, rapid, and clinically applicable tools to assess donkeys in acute pain under field conditions. Both scales showed high inter-observer reliability, which enables comparisons between different observers, and therefore also point towards a strong internal consistency of both scales. The EQUUS-DONKEY-COMPASS proved most useful to assess orthopaedic pain and pain after surgical castration, while the EQUUS-DONKEY-FAP proved most useful to assess acute colic and head-related pain in donkeys. In the future, blinded studies with for instance different analgesic treatment regimens could be conducted to further validate carefully selected EQUUS-DONKEY-FAP colic, orthopaedic, or surgical patients and their matched controls. 

## Figures and Tables

**Table 1:** Data of donkeys that were included in the study (*n* = 264).

Disease	Patient	Control	Sex: Gelding	Sex: Jenny	Sex: Jack	Age Mean Years (SD *)	Breed ^ Small	Breed ^ Average	Breed ^ Large
Head-related pain	18	39	45	12	0	14.1 (6.1)	12	43	2
Orthopaedic pain	25	68	55	38	0	18.4 (8.7)	7	79	7
Colic pain	12	28	30	9	1	19.8 (6.6)	3	37	0
Postoperative pain	24	50	46	17	11	9.9 (6.6)	6	64	4
Total	**79**	**185**	**176**	**76**	**12**	**15.6 (8.1)**	**28**	**223**	**13**

All animals were assessed on their arrival at The Donkey Sanctuary and those exhibiting extreme behaviours were excluded from the study beforehand. All control animals were assessed again to be pain-free before including them as control animals. * SD = standard deviation.; ^ Small donkeys: <90 cm; Average donkeys: 91–121 cm; and Large donkeys: >120 cm height at the withers. The bolds indicate the total values.

**Table 2:** The Equine Utrecht University Scale for Donkey Composite Pain Assessment (EQUUS-DONKEY-COMPASS) score table.

**Overall Appearance**	**Score**	**Pain Sounds**	**Score**
Alert and/or is interacting with companion/group	0	No audible signs of pain	0
Mildly “depressed” and/or restless and/or decreased interaction with group companion/group	1	Occasional teeth grinding or moaning (1 or 2 times/5 min)	1
Moderately “depressed” and/or aggressive or no reaction to companion/group	2	Frequent teeth grinding or moaning (3 or 4 times/5 min)	2
Severely “depressed”	3	Excessive teeth grinding or moaning (>4 times/5 min)	3
**Posture**	**Score**	**Changes in Behaviour of Companion/Group**	**Score**
Quietly standing and/or one hind leg resting	0	Patient is in the group	0
Slightly tucked up abdomen and/or mild weight shifting	1		
Extremely tucked up abdomen and/or hunched back and/or stretching limbs/body and/or mild muscle tremors	2		
Sits on hind quarters and/or extreme muscle tremors	3	Companion/group leaves or has left patient (excluding herd behaviour)	3
**Weight Distribution**	**Score**	**Eating (Present Food)**	**Score**
Normal weight distribution	0	Eats normally or fasts	0
		Eats less and/or slowly	2
Abnormal weight distribution	3	Not interested in food	3
**Laying down, Rolling (Excluding Auto Grooming)**	**Score**	**Movement**	**Score**
Does not lie down or rests lying down	0	No reluctance to move and normal gait	0
Attempts to lie down or is lying down <50% of the time	1	Mildly abnormal gait (1 or 2 out of 5 for lameness) and/or stiff walk	1
Lying down >50% of the time	2	Reluctance to move when motivated and/or severely abnormal gait (3 to 5 out of 5 for lameness)	2
Lies down in abnormal position: on its side with stretched limbs or on its back and/or is repeatedly rolling	3	No movement or is lying down	3
**Head Carriage**	**Score**	**Respiratory Rate**	**Score**
Ear base above withers or eats/drinks (from the ground)	0	12–28 breaths/min	0
		29–32 breaths/min	1
Ear base at the level of the withers	2	33–36 breaths/min	2
Ear base below the withers	3	>36 breaths/min	3
**Position of the Ears (>75% of the time)**	**Score**	**Reaction to Observer(s)**	**Score**
Normal position	0	Reaction to observer(s)	0
		Mild reaction to observer(s)	2
Abnormal position (backwards/sideways/flat)	3	No reaction to observer(s)	3
**Episodes of Tail Flicking (Excluding Flicking to Insects)**	**Score**	**Reaction to Palpation of the Painful Area**	**Score**
No tail flicking, tail in normal position	0	No reaction to palpation	0
Occasional tail flicking (1 or 2 episodes/5 min)	1		
Frequent tail flicking (3 or 4 episodes/5 min)	2	Mild reaction to palpation	2
Excessive tail flicking (>4 episodes/5 min) and/or lifts out tail or tail is tucked in	3	Severe reaction to palpation	3
**Kicking at Abdomen**	**Score**	**Heart Rate**	**Score**
Quietly standing, no kicking	0	32–52 beats/min	0
Looking at abdomen	1	53–60 beats/min	1
Lifting up hind legs, may kick once or twice at abdomen	2	61–68 beats/min	2
Extensive kicking at abdomen (>2 episodes /5 min)	3	>68 beats/min	3
**Pawing at Floor**	**Score**	**Rectal Temperature**	**Score**
Quietly standing, does not paw at floor	0	35.7–38.0 °C	0
Points limb	1	35.3–35.6 °C or 38.1–38.5 °C	1
Occasional pawing at floor (1 or 2 episodes/5 min)	2	34.7–35.2°C or 38.6–39.0 °C	2
Extensive pawing at floor (>2 episodes/5 min)	3	<34.6 °C or >39.1 °C	3
**Sweating**	**Score**	**Digestive Sounds**	**Score**
No signs of sweating	0	Normal motility	0
		Decreased motility	1
Signs of sweating (wet spots visible, no droplets or streams)	2	No motility	2
Excessive sweating (streams or droplets)	3	Hypermotility or steel band	3
**Total Composite Pain Score (Max Score = 60)**			**0–60**
Total scoring duration = 5 min			

**Table 3:** The Equine Utrecht University Scale for Donkey Facial Assessment of Pain (EQUUS-DONKEY-FAP) score table.

**Head**	**Score**	**Flehmen/Yawning/Smacking**	**Score**
Normal movement	0	Not seen	0
Less/no or more/ exaggerated movement	2	Seen	2
**Eyelids**	**Score**	**Ear Position**	**Score**
Opened	0	Normal position	0
More opened eyes or tightening of eyelids	1		
Obviously more opened eyes (sclera visible) or obvious orbital tightening of eyelids	2	Abnormal position (hang down/backwards)	2
**Focus**	**Score**	**Ear Response**	**Score**
Focused on environment	0	Clear response with both ears or ear closest to source	0
Less focused on environment	1	Delayed/reduced response to sounds	1
Not focused on environment	2	No response to sounds	2
**Nostrils**	**Score**	**Startle/Headshaking**	**Score**
Relaxed	0	No startle/headshaking	0
A bit more opened, nostrils lifted, wrinkles seen	1		
Obviously more opened, nostril flaring, possibly audible breathing	2	At least one startle (a sudden abrupt movement with the head as if suddenly aware of danger)/period of head shaking	2
**Corners Mouth/Lips**	**Score**	**Teeth Grinding and/or Moaning**	**Score**
Relaxed	0	Not been heard	0
Lifted	2	Heard	2
**Muscle Tone Head**	**Score**	**Sweating Behind the Ears**	**Score**
No fasciculations	0	No signs of sweating	0
Mild fasciculations	1		
Obvious fasciculations	2	Signs of sweating	2
**Total Donkey Facial Assessment of Pain Score (Max Score = 24)**			**0–24**
Total scoring duration = 2 min.			

**Table 4:** Sensitivity, specificity, positive and negative predictive value of the EQUUS-DONKEY-COMPASS and the EQUUS-DONKEY-FAP for different types of pain.

EQUUS-DONKEY	Sensitivity	Specificity	Positive Pred. Value	Negative Pred. Value
**COMPASS**				
Facial pain	16.7%	100%	100%	72.2%
Orthopaedic pain	88.0%	100%	100%	95.8%
Colic pain	83.3%	96.4%	90.9%	93.1%
Postoperative pain	20.8%	100%	100%	72.5%
**FAP**				
Facial pain	77.8%	89.7%	77.8%	89.7%
Orthopaedic pain	40%	91.2%	62.5%	80.5%
Colic pain	75%	96.4%	90%	90%
Postoperative pain	50%	96%	85.7%	80%

EQUUS-DONKEY—Equine University Utrecht Scale for Donkeys; COMPASS Composite Pain Assessment; FAP—Facial Assessment of Pain; Positive pred. value = positive predictive value, Negative pred. value—negative predictive value. Cut-off values that are used: ≥5 for EQUUS-DONKEY-COMPASS, ≥2 for EQUUS-DONKEY-FAP; Colic pain (*n* = 12 patients, *n* = 28 controls); Head-related pain (*n* = 18 patients, *n* = 39 controls); Orthopaedic pain (*n* = 25 patients, *n* = 68 controls); Postoperative pain (*n* = 24 patients, *n* = 50 controls).

**Table 5:** Weighting factors of individual parameters of EQUUS-DONKEY-COMPASS.

Weighting Factor for:	Facial Pain	Orthopaedic Pain	Colic Pain	Post-Op Pain
Overall appearance	1	1	3	0
Posture	0	0	2	0
Weight distribution	0	3	0	0
Laying down, rolling	0	0	0	0
Head carriage	0	0	2	0
Position of the ears (>75% of the time)	0	1	1	0
Episodes of tail flicking	0	0	0	0
Kicking at abdomen	0	0	0	0
Pawing at floor	0	1	0	0
Sweating	0	0	0	0
Pain sounds	0	0	0	0
Changes in behaviour of mate/group	0	0	1	0
Eating (present food)	0	0	2	0
Movement	0	3	1	0
Respiratory rate	0	0	0	0
Reaction to observer(s)	0	0	1	0
Reaction to palpation of the painful area	0	1	1	0
Heart rate	0	0	0	1
Rectal temperature	0	0	0	0
Digestive sounds	0	0	3	0

Where sensitivity or specificity is <25%, the weighting factor = 0; where it is 25%–50%, the weighting factor = 1; where it is 50–75%, the weighting factor = 2; when both sensitivity and specificity are ≥75%, the weighting factor = 3 (cut-off value for individual parameters was ≥1).

**Table 6:** Weighting factors of individual parameters of EQUUS-DONKEY-FAP.

Weighting Factor for:	Facial Pain	Orthopaedic Pain	Colic Pain	Post-Op Pain
Head	0	0	0	0
Eyelids	3	0	2	1
Focus	0	0	1	0
Nostrils	1	1	2	0
Corners mouth/lips	0	0	1	1
Muscle tone head	0	0	0	0
Flehming/yawning/smacking	0	0	0	0
Teeth grinding and/or moaning	0	0	0	0
Ear response	0	0	0	0
Ear position	0	1	1	0
Startle/headshaking	0	0	0	0
Sweating behind the ears	0	0	0	0

Where sensitivity or specificity is <25%, the weighting factor = 0; where it is 25–50%, the weighting factor = 1; where it is 50–75%, the weighting factor = 2; when both sensitivity and specificity are ≥75%, the weighting factor = 3 (cut-off value for individual parameters is ≥1.

## Supplementary Materials

The following files are available online at https://www.mdpi.com/2076-2615/10/2/354/s1. Table S1: Details on patient donkeys, Table S2: Details on control donkeys, Table S3: Age comparisons, Table S4: Donkey patient EQUUS-DONKEY-COMPASS scores, Table S5: Donkey control EQUUS-DONKEY-COMPASS scores, Table S6: Donkey patient EQUUS-DONKEY-FAP scores, Table S7: Donkey control EQUUS-DONKEY-FAP scores.

## References

[1] Ashley F.H., Waterman-Pearson A.E., Whay H.R. (2005). Behavioural Assessment of Pain in Horses and Donkeys: Application to Clinical Practice and Future Studies. Equine Vet. J..

[2] Robertson S. (2006). Overview—the Importance of Assessing Pain in Horses and Donkeys. Equine Vet. J..

[3] Gleerup K.B., Lindegaard C. (2016). Recognition and quantification of pain in horses: A tutorial review. Equine Vet. Educ..

[4] De Grauw J.C., van Loon J.P. (2016). Systematic pain assessment in horses. Vet. J..

[5] Van Loon J.P., van Dierendonck M.C. (2018). Objective pain assessment in horses (2014–2018). Vet. J..

[6] Pritchard J.C., Lindberg A.C., Main D.C.J., Whay H.R. (2005). Assessment of the welfare of working horses, mules and donkeys, using health and behaviour parameters. Prev. Vet. Med..

[7] Regan F.H., Hockenhull J., Pritchard J.C., Waterman-Pearson A.E., Whay H.R. (2014). Behavioural Repertoire of Working Donkeys and Consistency of Behaviour over Time, as a Preliminary Step towards Identifying Pain-Related Behaviours. PLoS ONE.

[8] Regan F.H., Hockenhull J., Pritchard J.C., Waterman-Pearson A.E., Whay H.R. (2016). Identifying behavioural differences in working donkeys in response to analgesic administration. Equine Vet. J..

[9] Burden F., Thiemann A. (2015). Donkeys Are Different. J. Equine Vet. Sci..

[10] Bussieres G., Jacques C., Lainay O., Beauchamp G., Leblond A., Cadore J.M., Desmaizieres M., Cuvelliez S.G., Troncy E. (2008). Development of a composite orthopaedic pain scale in horses. Res. Vet. Sci..

[11] Graubner C., Gerber V., Doherr M., Spadavecchia C. (2011). Clinical application and reliability of a post abdominal surgery pain assessment scale (PASPAS) in horses. Vet. J..

[12] Van Loon J.P., van Dierendonck M.C. (2015). Monitoring acute equine visceral pain with the Equine Utrecht University Scale for Composite Pain Assessment (EQUUS-COMPASS) and the Equine Utrecht University Scale for Facial Assessment of Pain (EQUUS-FAP): A scale-construction study. Vet. J..

[13] Dalla Costa E., Minero M., Lebelt D., Stucke D., Canali E., Leach M. (2014). Development of the Horse Grimace Scale (HGS) as a Pain Assessment Tool in Horses Undergoing Routine Castration. PLoS ONE.

[14] Gleerup K.B., Forkman B., Lindegaard C., Andersen P.H. (2015). An equine pain face. Vet. Anaesth. Analg..

[15] Grint N.J., Murrell J.C., Whay H.R. (2015). Investigating the opinions of donkey owners and veterinary surgeons towards pain and analgesia in donkeys. Equine Vet. Educ..

[16] VanDierendonck M.C., van Loon J.P. (2016). Monitoring acute equine visceral pain with the Equine Utrecht University Scale for Composite Pain Assessment (EQUUS-COMPASS) and the Equine Utrecht University Scale for Facial Assessment of Pain (EQUUS-FAP): A validation study. Vet. J..

[17] Svendsen E.D. (2008). The professional handbook of the donkey.

[18] Van Loon J.P., van Dierendonck M.C. (2017). Monitoring equine head-related pain with the Equine Utrecht University scale for facial assessment of pain (EQUUS-FAP). Vet. J..

[19] Van Loon J.P., van Dierendonck M.C. (2019). Pain assessment in horses after orthopaedic surgery and with orthopaedic trauma. Vet. J..

[20] Abass M.M., Rizk A.Z., Mosbah E.M., Zaghloul A.E. (2018). Anaesthetic and cardiopulmonary evaluation following xylazine-diazepam-ketamine-propofol administration with or without local infiltration analgesia using mepivacaine during inguinal castration in donkeys. Alex. J. Vet. Sci..

[21] Abass M., Picek S., Garzón J.F.G., Kÿhnle C., Zaghlou A., Bettschart-Wolfensberger R. (2018). Local mepivacaine before castration of horses under medetomidine isoflurane balanced anaesthesia is effective to reduce perioperative nociception and cytokine release. Equine Vet. J..

[22] Lindegaard C., Thomsen M.H., Larsen S., Andersen P.H. (2010). Analgesic efficacy of intra-articular morphine in experimentally induced radiocarpal synovitis in horses. Vet. Anaesth. Analg..

[23] Wathan J., Burrows A.M., Waller B.M., McComb K. (2015). EquiFACS: The Equine Facial Action Coding System. PLoS ONE.

[24] Tuyttens F.A.M., de Graaf S., Heerkens J.L.T., Jacobs L., Nalon E., Ott S., Stadig L., van Laer E., Ampe B. (2014). Observer bias in animal behaviour research: Can we believe what we score, if we score what we believe?. Anim. Behav..

[25] McLennan K.M. (2018). Why Pain Is Still a Welfare Issue for Farm Animals, and How Facial Expression Could Be the Answer. Agriculture.

