# Supplementary material for: Monitoring Acute Pain in Donkeys with the Equine Utrecht University Scale for Donkeys Composite Pain Assessment (EQUUS-DONKEY-COMPASS) and the Equine Utrecht University Scale for Donkey Facial Assessment of Pain (EQUUS-DONKEY-FAP)
Source: Animals (Basel). 2020 Feb 22;10(2):354. doi: 10.3390/ani10020354 (PMC7070438; doi:10.3390/ani10020354)
Supplement: Supplementary file 1 [file animals-10-00354-s001.zip › S3 Table age comparisons copy.pdf]

S3 Age normality, distribution and correlation with COMPASS and FAP scores

S3-1 Normality of the distribution of age in patients and in controls

Shapiro Wilks	Patients				Control				Patients + control		
	n	SW*	p		n	SW*	p		n	SW*	p
**All animals**	79	0.975	**0.122^**		185	0.967	0.0001		264	0.971	0.0001
**Head-related pain**	18	0.980	**0.950^**		39	0.969	**0.342^**		**57**	**0.982**	**0.546^**
**Orthopaedic pain**	25	0.955	**0.317^**		68	0.921	0.0001		93	0.935	0.0001
**Colic pain**	12	0.937	**0.465^**		28	0.928	**0.054^**		40	0.938	0.031
**Postoperative pain**	24	0.942	**0.180^**		50	0.928	0.007		74	0.935	0.001

* SW = Shapiro Wilks test

^ Normally distributed

S3-2 Comparison of the distribution of age between patients and controls

	Patients n	Control n		MWU*	
	n	n		U	p
**All animals**	79	185		7400.5	0.705
**Head-related pain**	18	39		382	0.587
**Orthopaedic pain**	25	68		825	0.828
**Colic pain**	12	28		155.5	0.718
**Postoperative pain**	24	50		578	0.864

* MWU = Mann Whitney U test

Only in Head related pain both patients and controls were normally distributed: independent t-test = -0.58, df = 55, p = 0.564.

S3-3 Correlation between age and CPS and between age and FAP in patients only

	Patients	Age - COMPASS		Age - FAP	
	n	Rho^	p	Rho^	p
**All patients**	79	0.34	0.002*	0.06	0.328
**Head-related pain**	18	0.23	0.353	-0.12	0.634
**Orthopaedic pain**	25	0.27	0.201	0.16	0.438
**Colic pain**	12	0.09	0.768	-0.03	0.939
**Postoperative pain**	24	-0.36	0.088	0.20	0.344

^ Spearman's rho

* Significant
