# Supplementary material for: Monitoring Acute Pain in Donkeys with the Equine Utrecht University Scale for Donkeys Composite Pain Assessment (EQUUS-DONKEY-COMPASS) and the Equine Utrecht University Scale for Donkey Facial Assessment of Pain (EQUUS-DONKEY-FAP)
Source: Animals (Basel). 2020 Feb 22;10(2):354. doi: 10.3390/ani10020354 (PMC7070438; doi:10.3390/ani10020354)
Supplement: Supplementary file 1 [file animals-10-00354-s001.zip › S6 Table donkey patients EQUUS DONKEY FAP scores.pdf]

S6 Donkey patients EQUUS DONKEY FAP scores

FAP		T=0a pre - ok		T=0b post - ok		T=1a morning		T=1b afternoon		T= 2a morning		T= 2b afternoon		T=3a morning		T=3b afternoon	
nr	Patient Donkey code	Obs1	Obs2	Obs1	Obs2	Obs1	Obs2	Obs1	Obs2	Obs1	Obs2	Obs1	Obs2	Obs1	Obs2	Obs1	Obs2
1	**PatA01**	0	1			3	0										
2	**PatA02**	7	4														
3	**PatA03**	2	1			2	2										
4	**PatA04**	0	0	3	2			0	3	3	1						
5	**PatA05**	2	4	2	2	0	1										
6	**PatA06**	3	4														
7	**PatA07**	0	3	1	0												
8	**PatA08**	1	3														
9	**PatA09**	1	1														
10	**PatA10**	9	9	5	8	7	8										
11	**PatA11**	0	1														
12	**PatA12**	2	1														
13	**PatA13**	4	3														
14	**PatA14**	8	7	7	4												
15	**PatA15**	8	5														
16	**PatA16**	11	10	2	1												
17	**PatA17**	3	5	2	3	0	0										
18	**PatA18**	1	1	0	1												
19	**PatA19**	11	8			6	9										
20	**PatB01**	1	0	0	0	1	0	0	0					0	0		
21	**PatB02**	0	0			0	0	0	0					0	0		
22	**PatB05**	2	2					0	0	0	0						
23	**PatB06**	2	1			0	0			0	0						
24	**PatB07**			0	0	0	0	0	0								
25	**PatB08**			0	0	0	0	0	0								
26	**PatB09**	0	0			0	0			0	0						
27	**PatB10**	5	4			0	0			0	0						
28	**PatB11**	0	0			0	0										
29	**PatB12**	2	2			0	0										
30	**PatB13**	1	1														
31	**PatB14**	1	0			0	0							0	0		
32	**PatB15**	5	4			0	0										
33	**PatB16**	1	1			0	0										
34	**PatB17**	3	5														
35	**PatB19**	2	0					0	0	1	1						
36	**PatB20**	8	5			3	1										
37	**PatB21**	4	3														
38	**PatB22**	0	0											0	0	0	0
39	**PatB23**	2	0											0	0		
40	**PatB24**	1	1			0	0					0	0				
41	**PatB25**	4	4			0	0			0	2						
42	**PatB26**	0	0			0	0			0	0						
43	**PatB27**	0	0											0	0		
44	**PatB28**	0	2							0	0						
45	**PatC01**	2	2	0	1	1	1										
46	**PatC02**	0	2	0	1	2	3										
47	**PatC03**	5	7	2	2	5	5										
48	**PatC04**					4	1	2	1	4	3	1	1	3	3	1	1
49	**PatC05**					2	1	1	1	0	0	0	0	0	0	0	0
50	**PatC06**					3	4	1	2	2	2	1	1	1	1	1	1
51	**PatC07**					3	3	3	3	3	3	3	3				
52	**PatC08**	1	1	5	5												
53	**PatC09**	0	0	2	3												
54	**PatC10**	0	0	0	0	1	1										
55	**PatC11**	0	0	0	0	3	3										
56	**PatC12**	0	0	4	4	2	0										
57	**PatC13**	0	0			1	1										
58	**PatC14**	5	5			4	4	6	6								
59	**PatC15**					5	5										
60	**PatC16**	0	1	0	0												
61	**PatC17**	2	2	1	1	0	0	0	0	2	2						
62	**PatC18**			4	4	3	2	3	3	1	1	4	4				
63	**PatC19**	1	1	4	4	1	2	1	1	1	1	3	3				
64	**PatC20**	4	4	2	2	4	4	2	2								
65	**PatC21**	2	2	2	2												
66	**PatC22**			4	4												
67	**PatC23**			4	4												
68	**PatC24**	1	1	2	4	1	2										
69	**PatC25**	1	1	5	5	2	2										
70	**PatC26**			3	3	2	2	2	2								

FAP		T=0a pre - ok		T=0b post - ok		T=1a morning		T=1b afternoon		T= 2a morning		T= 2b afternoon		T=3a morning		T=3b afternoon	
nr	Patient Donkey code	Obs1	Obs2	Obs1	Obs2	Obs1	Obs2	Obs1	Obs2	Obs1	Obs2	Obs1	Obs2	Obs1	Obs2	Obs1	Obs2
71	**PatC27**					3	3	2	2								
72	**PatC28**					2	2	1	1								
73	**PatC29**					5	5	3	3								
74	**PatC30**	0	1														
75	**PatC31**	6	6	2	1	0	0										
76	**PatC32**	4	4	1	3	1	3										
77	**PatC33**			2	2	4	4	2	2	1	1	0	0				
78	**PatC34**	0	0	2	2	2	2										
79	**PatC35**	1	2	6	6												

T0a = admission to clinic for facial pain, orthopaedic- and colic pain patients; Baseline assessment before surgery for surgery patients,
T0b = afternoon of first day after admission to clinic for facial pain, orthopaedic- and colic pain patients; First assessment 4 hours after surgery for surgical patients.
T1a = morning assessment of day 1
T1b = afternoon assessment of day 1.
T2a = morning assessment of day 2.
T2b = afternoon assessment of day 2.
T3a = morning assessment of day 3.
T3b = afternoon assessment of day 3.

Obs1, Obs2 = Observer 1 and Observer 2
