# Supplementary material for: Monitoring Acute Pain in Donkeys with the Equine Utrecht University Scale for Donkeys Composite Pain Assessment (EQUUS-DONKEY-COMPASS) and the Equine Utrecht University Scale for Donkey Facial Assessment of Pain (EQUUS-DONKEY-FAP)
Source: Animals (Basel). 2020 Feb 22;10(2):354. doi: 10.3390/ani10020354 (PMC7070438; doi:10.3390/ani10020354)
Supplement: Supplementary file 1 [file animals-10-00354-s001.zip › S5 Table donkey control EQUUS DONKEY COMPASS scores.pdf]

S5 Donkey control EQUUS DONKEY COMPASS score

COMPASS		T = 0a		T=0b	
nr	Control Donkey code	Obs1	Obs2	Obs1	Obs2
1	ConC01			0	0
2	ConC02			1	1
3	ConC03			0	0
4	ConC04			1	1
5	ConC05			0	0
6	ConC06			0	0
7	ConC07	0	0		
8	ConC08	0	0		
9	ConC09	0	1		
10	ConC10	0	0		
11	ConC11	0	0		
12	ConC12	0	0		
13	ConC13	0	0		
14	ConC14	0	0		
15	ConC15			0	0
16	ConC16			1	1
17	ConC17			0	0
18	ConC18			0	0
19	ConC19			0	0
20	ConC20			0	0
21	ConC21	0	0		
22	ConC22	0	0		
23	ConC23	0	0		
24	ConC24	0	0		
25	ConC25	0	0		
26	ConC26	0	0		
27	ConC27	0	0		
28	ConC28	0	0		
29	ConC29	0	0		
30	ConC30	0	0		
31	ConC31			0	0
32	ConC32			0	0
33	ConC33	0	0		
34	ConC34	0	0		
35	ConC35	0	0		
36	ConC36	0	0		
37	ConC37	0	0		
38	ConC38	0	0		
39	ConC39	0	0		
40	ConC40	0	0		
41	ConC41	0	0		
42	ConC42	0	0		
43	ConC43	0	0		
44	ConC44	0	0		
45	ConC45	0	0		
46	ConC46	0	0		
47	ConC47			0	0
48	ConC48			0	0
49	ConC49			0	0
50	ConC50			0	0
51	ConC51	0	0		
52	ConC52	0	0		
53	ConC53	0			
54	ConC54	0	0		
55	ConC55	0	0		
56	ConC56	0	0		
57	ConC57	0	0		
58	ConC58	0	0		
59	ConC59	0	0		
60	ConC60	0	0		
61	ConC61			0	0
62	ConC62	0	0		
63	ConC63			0	0

COMPASS		T = 0a		T=0b	
nr	Control Donkey code	Obs1	Obs2	Obs1	Obs2
64	ConC64			0	0
65	ConC65	0	0		
66	ConC66	0	0		
67	ConC67			0	0
68	ConC68			0	0
69	ConC69			0	0
70	ConC70			0	0
71	ConB01	3	3		
72	ConB02	2	2		
73	ConB03	1	1		
74	ConB04	0	0		
75	ConB05		0		
76	ConB06	1	3		
77	ConB13	0	0		
78	ConB14	0	0		
79	ConB15	2	2		
80	ConB16	3	4		
81	ConB17	0	0		
82	ConB18	1	3		
83	ConB19	3	3		
84	ConB20	0	0		
85	ConB21	0	0		
86	ConB22	0	0		
87	ConB23	0	0		
88	ConB24		0		
89	ConB25	0	0		
90	ConB26	2	3		
91	ConB27		0		
92	ConB28	0	0		
93	ConB29	0	0		
94	ConB30	0	0		
95	ConB31	0	0		
96	ConB32	0	0		
97	ConB33	0	0		
98	ConB34	0	0		
99	ConB35	0	0		
100	ConB36	3	3		
101	ConB37	0	0		
102	ConB38	0	0		
103	ConB39	0	1		
104	ConB40	1	1		
105	ConB41	1	1		
106	ConB42	0	0		
107	ConB46	0	0		
108	ConB47	0	0		
109	ConB48	0	0		
110	ConB49	0	0		
111	ConB50	0	0		
112	ConB51	0	0		
113	ConB52	0	0		
114	ConB53	0	0		
115	ConB54	0	0		
116	ConB55	0	0		
117	ConB56	0	0		
118	ConB57	0	0		
119	ConB58	0	0		
120	ConB59	0	0		
121	ConB60	0	0		
122	ConB61	0	0		
123	ConB62	0	0		
124	ConB63	0	0		
125	ConB64	0	0		
126	ConB65	0	0		
127	ConB66	0	0		
128	ConB67	0	0		
129	ConB68	0	0		
130	ConB69	0	0		
131	ConB70	0	0		

COMPASS		T = 0a		T=0b	
nr	Control Donkey code	Obs1	Obs2	Obs1	Obs2
132	**ConB71**	0	0		
133	**ConB72**	0	0		
134	**ConB73**	0	0		
135	**ConB74**	0	0		
136	**ConB75**	0	0		
137	**ConA01**	0	0		
138	**ConA02**	0	0		
139	**ConA03**	0	0		
140	**ConA04**	1	1		
141	**ConA05**	3	0		
142	**ConA06**	0	0		
143	**ConA07**	3	5		
144	**ConA08**	0	0		
145	**ConA09**	0	0		
146	**ConA10**	0	0		
147	**ConA11**	3	0		
148	**ConA12**	0	0		
149	**ConA13**	4	1		
150	**ConA14**	1	1		
151	**ConA15**	2	2		
152	**ConA16**	4	3		
153	**ConA17**	1	5		
154	**ConA18**	4	1		
155	**ConA19**	0	1		
156	**ConA20**	1	1		
157	**ConA21**	1	1		
158	**ConA22**	1	0		
159	**ConA23**	1	0		
160	**ConA24**	1	1		
161	**ConA25**	0	0		
162	**ConA26**	5	5		
163	**ConA27**	0	0		
164	**ConA28**	1	0		
165	**ConA29**	2	3		
166	**ConA30**	2	3		
167	**ConA31**	3	3		
168	**ConA32**	4	5		
169	**ConA33**	3	4		
170	**ConA34**	4	2		
171	**ConA35**	0	0		
172	**ConA36**	3	1		
173	**ConA37**	2	0		
174	**ConA38**	1	1		
175	**ConA39**	2	1		
176	**ConA40**	3	3		
177	**ConA41**	1	1		
178	**ConA42**	2	2		
179	**ConA43**	2	2		
180	**ConA44**	2	2		
181	**ConA45**	0	0		
182	**ConA46**	0	0		
183	**ConA47**	1	1		
184	**ConA48**	2	0		
185	**ConA49**	0	0		

T0a = Baseline assessment
T0b = Afternoon of first day (used for donkeys who were control for operation patiens if available)

Obs1, Obs2 = Observer 1 and Observer 2
