# Supplementary material for: Monitoring Acute Pain in Donkeys with the Equine Utrecht University Scale for Donkeys Composite Pain Assessment (EQUUS-DONKEY-COMPASS) and the Equine Utrecht University Scale for Donkey Facial Assessment of Pain (EQUUS-DONKEY-FAP)
Source: Animals (Basel). 2020 Feb 22;10(2):354. doi: 10.3390/ani10020354 (PMC7070438; doi:10.3390/ani10020354)
Supplement: Supplementary file 1 [file animals-10-00354-s001.zip › S4 Table donkey patients EQUUS DONKEY COMPASS scores.pdf]

S4 Donkey patients EQUUS DONKEY COMPASS scores

COMPASS		T=0a pre - ok		T=0b post - ok		T=1a morning		T=1b afternoon		T= 2a morning		T= 2b afternoon		T=3a morning		T=3b afternoon	
nr	Patient Donkey code	Obs1	Obs2	Obs1	Obs2	Obs1	Obs2	Obs1	Obs2	Obs1	Obs2	Obs1	Obs2	Obs1	Obs2	Obs1	Obs2
1	**PatA01**	10	9			6	3										
2	**PatA02**	13	13														
3	**PatA03**	3	2			0	0										
4	**PatA04**	2	2	4	0			2	1	1	0						
5	**PatA05**	5	5	8	6	6	4										
6	**PatA06**	7	9														
7	**PatA07**	2	4	1	3												
8	**PatA08**	8	11														
9	**PatA09**	7	11														
10	**PatA10**	19	22	5	10	7	8										
11	**PatA11**	6	10														
12	**PatA12**	10	16														
13	**PatA13**	8	9														
14	**PatA14**	2	4	0	0												
15	**PatA15**	11	14														
16	**PatA16**	24	16	6	5												
17	**PatA17**	7	5	1	1	5	5										
18	**PatA18**	4	2	3	6												
19	**PatA19**	18	16			13	11										
20	**PatB01**	8	8	1	1	4	4	1	1					2	4		
21	**PatB02**	1	0			1	1	0	0					4	2		
22	**PatB05**	3	5					2	1	2	2						
23	**PatB06**	9	11			0	0			3	1						
24	**PatB07**			6	6	2	2	0	0								
25	**PatB08**			6	6	5	5	0	0								
26	**PatB09**	7	7			4	5			0	0						
27	**PatB10**	17	13			0	0			0	0						
28	**PatB11**	2	2			3	3										
29	**PatB12**	1	0			0	0										
30	**PatB13**	0	0														
31	**PatB14**	6	5			0	0							2	2		
32	**PatB15**	15	14			8	7										
33	**PatB16**	9	9			6	5										
34	**PatB17**	9	11														
35	**PatB19**	8	4					5	5	6	6						
36	**PatB20**	6	6			0	0										
37	**PatB21**	17	15														
38	**PatB22**	7	8											7	7	10	10
39	**PatB23**	10	5											0	0		
40	**PatB24**	2	2			0	0					0	0				
41	**PatB25**	16	16			0	0			0	3						
42	**PatB26**	6	6			0	0			0	0						
43	**PatB27**	6	6											0	0		
44	**PatB28**	4	1							0	0						
45	**PatC01**	1	2	6	5	2	4										
46	**PatC02**	0	1	0	0	0	0										
47	**PatC03**	4	4	2	1	2	0										
48	**PatC04**					2	1	1	0	2	2	0	0	2	1	0	0
49	**PatC05**					0	0	0	0	0	0	0	0	0	0	0	0
50	**PatC06**					1	0	0	0	0	0	0	0	0	0	3	2
51	**PatC07**					5	3	1	1	1	1	1	1				
52	**PatC08**	0	0	0	1												
53	**PatC09**	0	0	2	1												
54	**PatC10**	0	0	0	0	0	0										
55	**PatC11**	1	1	1	1	4	4										
56	**PatC12**	0	0	3	2	1	1										
57	**PatC13**	0	0			1	1										
58	**PatC14**	10	10			3	3	5	5								
59	**PatC15**					7	7										
60	**PatC16**	0	0	1	1												
61	**PatC17**	0	0	0	0	0	0	0	0	0	0						
62	**PatC18**			9	9	2	2	5	5	0	0	1	1				
63	**PatC19**	0	0	5	5	0	0	0	0	0	0	0	0				
64	**PatC20**	3	3	2	2	2	3	2	2								
65	**PatC21**	3	3	2	3												
66	**PatC22**			10	10												
67	**PatC23**			10	10												
68	**PatC24**	0	0	0	0	0	0										
69	**PatC25**	0	0	1	1	0	0										
70	**PatC26**			0	0	1	1	1	2								

COMPASS		T=0a pre - ok		T=0b post - ok		T=1a morning		T=1b afternoon		T= 2a morning		T= 2b afternoon		T=3a morning		T=3b afternoon	
**nr**	**Patient Donkey code**	**Obs1**	**Obs2**	**Obs1**	**Obs2**	**Obs1**	**Obs2**	**Obs1**	**Obs2**	**Obs1**	**Obs2**	**Obs1**	**Obs2**	**Obs1**	**Obs2**	**Obs1**	**Obs2**
71	**PatC27**					2	2	0	0								
72	**PatC28**					0	1	0	0								
73	**PatC29**					2	2	1	1								
74	**PatC30**	5	5														
75	**PatC31**	2	3	0	0	0	0										
76	**PatC32**	1	1	0	0	0	0										
77	**PatC33**			4	4	0	0	1	1	0	0	0	0				
78	**PatC34**	0	0	1	1	0	0										
79	**PatC35**	1	1	1	1												

T0a = admission to clinic for facial pain, orthopaedic- and colic pain patients; Baseline assessment before surgery for surgery patients,
T0b = afternoon of first day after admission to clinic for facial pain, orthopaedic- and colic pain patients; First assessment 4 hours after surgery for surgical patients.
T1a = morning assessment of day 1
T1b = afternoon assessment of day 1.
T2a = morning assessment of day 2.
T2b = afternoon assessment of day 2.
T3a = morning assessment of day 3.
T3b = afternoon assessment of day 3.

Obs1, Obs2 = Observer 1 and Observer 2
