# Supplementary material for: Monitoring Acute Pain in Donkeys with the Equine Utrecht University Scale for Donkeys Composite Pain Assessment (EQUUS-DONKEY-COMPASS) and the Equine Utrecht University Scale for Donkey Facial Assessment of Pain (EQUUS-DONKEY-FAP)
Source: Animals (Basel). 2020 Feb 22;10(2):354. doi: 10.3390/ani10020354 (PMC7070438; doi:10.3390/ani10020354)
Supplement: Supplementary file 1 [file animals-10-00354-s001.zip › S7 Table donkey control EQUUS DONKEY FAP scores.pdf]

S7 Donkey control EQUUS DONKEY FAP scores

FAP		T = 0a		T=0b	
nr	Control Donkey code	Obs1	Obs2	Obs1	Obs2
1	ConC01			4	4
2	ConC02			0	0
3	ConC03			0	0
4	ConC04			0	0
5	ConC05			0	0
6	ConC06			0	0
7	ConC07	0	0		
8	ConC08	0	0		
9	ConC09	2	0		
10	ConC10	0	0		
11	ConC11	0	0		
12	ConC12	0	0		
13	ConC13	0	0		
14	ConC14	0	0		
15	ConC15			0	0
16	ConC16			1	1
17	ConC17			0	0
18	ConC18			0	0
19	ConC19			0	0
20	ConC20			0	0
21	ConC21	0	1		
22	ConC22	0	0		
23	ConC23	0	0		
24	ConC24	0	0		
25	ConC25	0	0		
26	ConC26	0	0		
27	ConC27	0	0		
28	ConC28	0	0		
29	ConC29	0	0		
30	ConC30	0	0		
31	ConC31			0	0
32	ConC32			0	2
33	ConC33	2	2		
34	ConC34	0	0		
35	ConC35	2	0		
36	ConC36	1	1		
37	ConC37	0	0		
38	ConC38	0	0		
39	ConC39	0	0		
40	ConC40	0	0		
41	ConC41	0	0		
42	ConC42	0	0		
43	ConC43	0	0		
44	ConC44	0	0		
45	ConC45	0	0		
46	ConC46	0	0		
47	ConC47			1	1
48	ConC48			0	0
49	ConC49			1	1
50	ConC50			0	0
51	ConC51	0	0		
52	ConC52	0	0		
53	ConC53	0	0		
54	ConC54	0	0		
55	ConC55	0	0		
56	ConC56	0	0		
57	ConC57	0	0		
58	ConC58	0	0		
59	ConC59	0	0		
60	ConC60	0	0		
61	ConC61			2	2
62	ConC62	0	0		
63	ConC63			1	1
64	ConC64			0	0

FAP		T = 0a		T=0b	
nr	Control Donkey code	Obs1	Obs2	Obs1	Obs2
65	**ConC65**	0	0		
66	**ConC66**	0	0		
67	**ConC67**			1	1
68	**ConC68**			0	0
69	**ConC69**			0	0
70	**ConC70**			0	0
71	**ConB01**	2	2		
72	**ConB02**	0	0		
73	**ConB03**	0	0		
74	**ConB04**	0	0		
75	**ConB05**		0		
76	**ConB06**	0	0		
77	**ConB13**	0	0		
78	**ConB14**	0	0		
79	**ConB15**	0	1		
80	**ConB16**	0	0		
81	**ConB17**	0	0		
82	**ConB18**	0	0		
83	**ConB19**	0	0		
84	**ConB20**	0	0		
85	**ConB21**	0	0		
86	**ConB22**	0	0		
87	**ConB23**	0	0		
88	**ConB24**		0		
89	**ConB25**	2	2		
90	**ConB26**	0	2		
91	**ConB27**		0		
92	**ConB28**	0	0		
93	**ConB29**	0	0		
94	**ConB30**	0	0		
95	**ConB31**	0	0		
96	**ConB32**	0	0		
97	**ConB33**	0	0		
98	**ConB34**	0	0		
99	**ConB35**	0	0		
100	**ConB36**	0	0		
101	**ConB37**	0	0		
102	**ConB38**	0	0		
103	**ConB39**	0	0		
104	**ConB40**	0	0		
105	**ConB41**	0	0		
106	**ConB42**	0	0		
107	**ConB46**	0	0		
108	**ConB47**	0	0		
109	**ConB48**	0	0		
110	**ConB49**	0	0		
111	**ConB50**	0	0		
112	**ConB51**	0	0		
113	**ConB52**	0	0		
114	**ConB53**	2	2		
115	**ConB54**	0	0		
116	**ConB55**	0	0		
117	**ConB56**	0	0		
118	**ConB57**	0	0		
119	**ConB58**	0	0		
120	**ConB59**	0	0		
121	**ConB60**	0	0		
122	**ConB61**	0	0		
123	**ConB62**	0	0		
124	**ConB63**	0	0		
125	**ConB64**	0	0		
126	**ConB65**	0	0		
127	**ConB66**	0	0		
128	**ConB67**	0	0		
129	**ConB68**	0	0		
130	**ConB69**	0	0		
131	**ConB70**	0	0		
132	**ConB71**	0	0		

FAP		T = 0a		T=0b	
nr	Control Donkey code	Obs1	Obs2	Obs1	Obs2
133	**ConB72**	0	0		
134	**ConB73**	0	0		
135	**ConB74**	0	0		
136	**ConB75**	0	0		
137	**ConA01**	0	0		
138	**ConA02**	0	0		
139	**ConA03**	2	0		
140	**ConA04**	1	1		
141	**ConA05**	3	1		
142	**ConA06**	0	3		
143	**ConA07**	0	0		
144	**ConA08**	0	0		
145	**ConA09**	1	1		
146	**ConA10**	0	1		
147	**ConA11**	0	1		
148	**ConA12**	0	0		
149	**ConA13**	0	0		
150	**ConA14**	0	0		
151	**ConA15**	0	0		
152	**ConA16**	0	0		
153	**ConA17**	0	1		
154	**ConA18**	1	2		
155	**ConA19**	1	1		
156	**ConA20**	1	0		
157	**ConA21**	0	0		
158	**ConA22**	2	3		
159	**ConA23**	1	1		
160	**ConA24**	2	0		
161	**ConA25**	0	0		
162	**ConA26**	2	0		
163	**ConA27**	0	1		
164	**ConA28**	2	1		
165	**ConA29**	2	1		
166	**ConA30**	0	0		
167	**ConA31**	2	5		
168	**ConA32**	2	1		
169	**ConA33**	0	0		
170	**ConA34**	0	0		
171	**ConA35**	0	0		
172	**ConA36**	0	1		
173	**ConA37**	1	1		
174	**ConA38**	4	2		
175	**ConA39**	4	2		
176	**ConA40**	4	2		
177	**ConA41**	4	4		
178	**ConA42**	4	6		
179	**ConA43**	0	2		
180	**ConA44**	0	0		
181	**ConA45**	1	1		
182	**ConA46**	2	0		
183	**ConA47**	0	1		
184	**ConA48**	1	0		
185	ConA49	2	1		

T0a = Baseline assessment
T0b = Afternoon of first day (used for donkeys who were control for operation patiens if available)

Obs1, Obs2 = Observer 1 and Observer 2
