# Supplementary material for: Monitoring Acute Pain in Donkeys with the Equine Utrecht University Scale for Donkeys Composite Pain Assessment (EQUUS-DONKEY-COMPASS) and the Equine Utrecht University Scale for Donkey Facial Assessment of Pain (EQUUS-DONKEY-FAP)
Source: Animals (Basel). 2020 Feb 22;10(2):354. doi: 10.3390/ani10020354 (PMC7070438; doi:10.3390/ani10020354)
Supplement: Supplementary file 1 [file animals-10-00354-s001.zip › S2 Table details control donkeys.pdf]

## S2 Details Donkey controls

| nr | Control Donkey code | sex | age | size* |
|---|---|---|---|---|
| 1 | **ConA01** | gelding | 8 | average |
| 2 | **ConA02** | gelding | 9 | large |
| 3 | **ConA03** | gelding | 8 | average |
| 4 | **ConA04** | Jenny | 26 | average |
| 5 | **ConA05** | Jenny | 20 | average |
| 6 | **ConA06** | Jenny | 30 | average |
| 7 | **ConA07** | Jenny | 9 | smal |
| 8 | **ConA08** | gelding | 9 | average |
| 9 | **ConA09** | gelding | 8 | average |
| 10 | **ConA10** | Jenny | 10 | average |
| 11 | **ConA11** | Jenny | 12 | large |
| 12 | **ConA12** | Jenny | 22 | average |
| 13 | **ConA13** | Jenny | 6 | average |
| 14 | **ConA14** | Jenny | 6 | average |
| 15 | **ConA15** | Jenny | 2 | average |
| 16 | **ConA16** | gelding | 22 | average |
| 17 | **ConA17** | Jenny | 26 | smal |
| 18 | **ConA18** | gelding | 32 | average |
| 19 | **ConA19** | gelding | 17 | large |
| 20 | **ConA20** | gelding | 26 | average |
| 21 | **ConA21** | Jenny | 12 | average |
| 22 | **ConA22** | Jenny | 12 | average |
| 23 | **ConA23** | Jenny | 16 | average |
| 24 | **ConA24** | Jenny | 29 | average |
| 25 | **ConA25** | Jenny | 20 | smal |
| 26 | **ConA26** | Jenny | 26 | smal |
| 27 | **ConA27** | gelding | 6 | average |
| 28 | **ConA28** | gelding | 6 | average |
| 29 | **ConA29** | gelding | 6 | average |
| 30 | **ConA30** | Jenny | 14 | smal |
| 31 | **ConA31** | Jenny | 6 | average |
| 32 | **ConA32** | Jenny | 16 | smal |
| 33 | **ConA33** | gelding | 14 | smal |
| 34 | **ConA34** | gelding | 14 | average |
| 35 | **ConA35** | gelding | 12 | average |
| 36 | **ConA36** | gelding | 6 | average |
| 37 | **ConA37** | gelding | 9 | average |
| 38 | **ConA38** | Jack | 3 | average |
| 39 | **ConA39** | gelding | 14 | smal |
| 40 | **ConA40** | gelding | 17 | smal |
| 41 | **ConA41** | gelding | 16 | smal |
| 42 | **ConA42** | gelding | 11 | smal |
| 43 | **ConA43** | Jenny | 28 | smal |
| 44 | **ConA44** | gelding | 29 | average |
| 45 | **ConA45** | gelding | 26 | average |
| 46 | **ConA46** | gelding | 11 | average |
| 47 | **ConA47** | gelding | 22 | average |
| 48 | **ConA48** | gelding | 7 | average |
| 49 | **ConA49** | gelding | 9 | large |
| 50 | **ConB01** | Jenny | 19 | average |
| 51 | **ConB02** | Jenny | 24 | average |
| 52 | **ConB03** | Jenny | 21 | average |
| 53 | **ConB04** | Jenny | 30 | average |
| 54 | **ConB05** | Jenny | 11 | average |
| 55 | **ConB06** | Jenny | 23 | average |
| 56 | **ConB13** | gelding | 21 | average |
| 57 | **ConB14** | gelding | 24 | average |
| 58 | **ConB15** | gelding | 22 | average |
| 59 | **ConB16** | gelding | 31 | average |
| 60 | **ConB17** | gelding | 30 | average |
| 61 | **ConB18** | gelding | 18 | average |
| 62 | **ConB19** | Jenny | 0 | smal |

| nr | Control Donkey code | sex | age | size* |
|---|---|---|---|---|
| 63 | **ConB20** | gelding | 2 | average |
| 64 | **ConB21** | Jenny | 1 | smal |
| 65 | **ConB22** | Jenny | 4 | average |
| 66 | **ConB23** | Jenny | 1 | smal |
| 67 | **ConB24** | Jenny | 28 | average |
| 68 | **ConB25** | gelding | 22 | average |
| 69 | **ConB26** | gelding | 25 | average |
| 70 | **ConB27** | Jenny | 14 | average |
| 71 | **ConB28** | Jenny | 16 | large |
| 72 | **ConB29** | Jenny | 7 | large |
| 73 | **ConB30** | gelding | 8 | average |
| 74 | **ConB31** | gelding | 10 | large |
| 75 | **ConB32** | gelding | 8 | average |
| 76 | **ConB33** | gelding | 19 | average |
| 77 | **ConB34** | gelding | 27 | average |
| 78 | **ConB35** | gelding | 16 | average |
| 79 | **ConB36** | gelding | 24 | average |
| 80 | **ConB37** | gelding | 17 | average |
| 81 | **ConB38** | gelding | 17 | average |
| 82 | **ConB39** | gelding | 35 | average |
| 83 | **ConB40** | gelding | 32 | average |
| 84 | **ConB41** | gelding | 34 | average |
| 85 | **ConB42** | Jenny | 26 | average |
| 86 | **ConB46** | Jenny | 6 | average |
| 87 | **ConB47** | gelding | 7 | average |
| 88 | **ConB48** | gelding | 6 | average |
| 89 | **ConB49** | gelding | 18 | average |
| 90 | **ConB50** | gelding | 14 | average |
| 91 | **ConB51** | gelding | 14 | average |
| 92 | **ConB52** | gelding | 31 | average |
| 93 | **ConB53** | gelding | 26 | average |
| 94 | **ConB54** | gelding | 19 | average |
| 95 | **ConB55** | gelding | 26 | average |
| 96 | **ConB56** | gelding | 16 | average |
| 97 | **ConB57** | Jenny | 8 | average |
| 98 | **ConB58** | Jenny | 17 | average |
| 99 | **ConB59** | gelding | 21 | average |
| 100 | **ConB60** | gelding | 5 | average |
| 101 | **ConB61** | gelding | 9 | average |
| 102 | **ConB62** | gelding | 9 | average |
| 103 | **ConB63** | gelding | 18 | average |
| 104 | **ConB64** | gelding | 18 | average |
| 105 | **ConB65** | gelding | 19 | average |
| 106 | **ConB66** | gelding | 23 | average |
| 107 | **ConB67** | gelding | 23 | average |
| 108 | **ConB68** | gelding | 23 | average |
| 109 | **ConB69** | gelding | 27 | average |
| 110 | **ConB70** | gelding | 26 | average |
| 111 | **ConB71** | gelding | 25 | average |
| 112 | **ConB72** | Jenny | 9 | average |
| 113 | **ConB73** | Jenny | 9 | average |
| 114 | **ConB74** | Jenny | 9 | average |
| 115 | **ConB75** | gelding | 21 | average |
| 116 | **ConC01** | Jack | 13 | average |
| 117 | **ConC02** | Jack | 5 | average |
| 118 | **ConC03** | gelding | 3 | average |
| 119 | **ConC04** | Jack | 15 | average |
| 120 | **ConC05** | gelding | 8 | average |
| 121 | **ConC06** | Jack | 7 | average |
| 122 | **ConC07** | gelding | 18 | average |
| 123 | **ConC08** | gelding | 8 | average |
| 124 | **ConC09** | Jenny | 19 | average |
| 125 | **ConC10** | gelding | 23 | average |
| 126 | **ConC11** | gelding | 13 | average |
| 127 | **ConC12** | gelding | 8 | average |
| 128 | **ConC13** | Jenny | 8 | smal |

| nr | Control Donkey code | sex | age | size* |
|---|---|---|---|---|
| 129 | **ConC14** | Jenny | 8 | smal |
| 130 | **ConC15** | gelding | 22 | average |
| 131 | **ConC16** | gelding | 22 | average |
| 132 | **ConC17** | gelding | 10 | average |
| 133 | **ConC18** | gelding | 9 | average |
| 134 | **ConC19** | gelding | 3 | average |
| 135 | **ConC20** | gelding | 4 | average |
| 136 | **ConC21** | gelding | 15 | average |
| 137 | **ConC22** | gelding | 14 | average |
| 138 | **ConC23** | gelding | 15 | average |
| 139 | **ConC24** | gelding | 14 | average |
| 140 | **ConC25** | gelding | 8 | average |
| 141 | **ConC26** | gelding | 5 | average |
| 142 | **ConC27** | gelding | 15 | average |
| 143 | **ConC28** | gelding | 14 | average |
| 144 | **ConC29** | gelding | 21 | average |
| 145 | **ConC30** | gelding | 24 | average |
| 146 | **ConC31** | gelding | 24 | average |
| 147 | **ConC32** | Jenny | 15 | average |
| 148 | **ConC33** | gelding | 8 | average |
| 149 | **ConC34** | gelding | 15 | average |
| 150 | **ConC35** | gelding | 22 | average |
| 151 | **ConC36** | Jenny | 24 | average |
| 152 | **ConC37** | gelding | 10 | average |
| 153 | **ConC38** | gelding | 10 | average |
| 154 | **ConC39** | Jenny | 13 | average |
| 155 | **ConC40** | Jenny | 18 | average |
| 156 | **ConC41** | gelding | 14 | smal |
| 157 | **ConC42** | gelding | 16 | average |
| 158 | **ConC43** | gelding | 28 | average |
| 159 | **ConC44** | Jenny | 29 | average |
| 160 | **ConC45** | gelding | 20 | average |
| 161 | **ConC46** | gelding | 19 | average |
| 162 | **ConC47** | gelding | 3 | average |
| 163 | **ConC48** | gelding | 15 | average |
| 164 | **ConC49** | Jenny | 6 | average |
| 165 | **ConC50** | Jenny | 7 | average |
| 166 | **ConC51** | gelding | 10 | average |
| 167 | **ConC52** | gelding | 10 | average |
| 168 | **ConC53** | gelding | 17 | average |
| 169 | **ConC54** | gelding | 19 | average |
| 170 | **ConC55** | Jenny | 4 | average |
| 171 | **ConC56** | gelding | 3 | average |
| 172 | **ConC57** | gelding | 9 | average |
| 173 | **ConC58** | gelding | 8 | average |
| 174 | **ConC59** | gelding | 23 | average |
| 175 | **ConC60** | Jenny | 22 | average |
| 176 | **ConC61** | Jenny | 9 | average |
| 177 | **ConC62** | Jenny | 9 | average |
| 178 | **ConC63** | gelding | 22 | average |
| 179 | **ConC64** | gelding | 14 | average |
| 180 | **ConC65** | gelding | 13 | average |
| 181 | **ConC66** | gelding | 13 | average |
| 182 | **ConC67** | gelding | 10 | average |
| 183 | **ConC68** | gelding | 9 | average |
| 184 | **ConC69** | gelding | 7 | average |
| 185 | **ConC70** | gelding | 7 | average |

*Small donkeys: <90 cm; Average donkeys: 91 - 121 cm;
Large donkeys: >120 cm height at the withers

| | |
|---|---|
| ConB07-ConB09 | Deleted from data set due to removal of related patie |
| ConB10-ConB12 | Deleted from data set due to removal of related patie |
| ConB43-ConB45 | Deleted from data set due to removal of related patie |
