# Supplementary material for: Monitoring Acute Pain in Donkeys with the Equine Utrecht University Scale for Donkeys Composite Pain Assessment (EQUUS-DONKEY-COMPASS) and the Equine Utrecht University Scale for Donkey Facial Assessment of Pain (EQUUS-DONKEY-FAP)
Source: Animals (Basel). 2020 Feb 22;10(2):354. doi: 10.3390/ani10020354 (PMC7070438; doi:10.3390/ani10020354)
Supplement: Supplementary file 1 [file animals-10-00354-s001.zip › S1 Table details patient donkeys.pdf]

S1 Details Donkey patients

nr	Patient Donkey code	Sex	Age	Size*	General diagnose before categorisation	Disease category		name control donkey 1	type of control	name control donkey 2	type of control	name control donkey 3	type of control
1	**PatA01**	gelding	7	average	Lameness unspecified	Orthopaedic pain		ConA01	herd	ConA02	herd	ConA03	herd
2	**PatA02**	Jenny	16	average	Solar abcess	Orthopaedic pain		ConA04	herd	ConA05	herd	ConA06	herd
3	**PatA03**	Jenny	8	smal	Multiple eye ulcerations	Head related pain		ConA07	herd	ConA08	herd	ConA09	herd
4	**PatA04**	gelding	9	average	Sarcoid removal	Post operative pain		ConA11	partner	ConA10	partner		
5	**PatA05**	Jack	1	smal	Castration	Post operative pain		ConA13	partner	ConA15	herd	ConA14	herd
6	**PatA06**	Jenny	29	average	Unspecified colic	Colic pain		ConA12	partner	ConA16	herd	ConA17	herd
7	**PatA07**	gelding	23	average	Dental extraction	Post operative pain		ConA18	herd	ConA19	herd	ConA20	herd
8	**PatA08**	Jenny	25	average	Solar abcess	Orthopaedic pain		ConA21	herd	ConA22	herd	ConA23	herd
9	**PatA09**	gelding	9	average	Solar abcess	Orthopaedic pain		ConA47	herd	ConA48	herd	ConA49	herd
10	**PatA10**	Jenny	26	average	Unspecified colic	Colic pain		ConA24	herd	ConA25	herd	ConA26	herd
11	**PatA11**	Jenny	8	average	Laminitic / WLD^	Orthopaedic pain		ConA27	herd	ConA28	herd	ConA29	herd
12	**PatA12**	gelding	17	smal	Lameness unspecified	Orthopaedic pain		ConA32	herd	ConA33	herd	ConA34	herd
13	**PatA13**	Jenny	5	average	Solar abcess	Orthopaedic pain		ConA30	herd	ConA31	herd		
14	**PatA14**	gelding	13	smal	Injured cornea	Head related pain		ConA40	herd	ConA41	herd	ConA42	herd
15	**PatA15**	gelding	13	smal	Lameness unspecified	Orthopaedic pain		ConA36	partner	ConA35	herd	ConA37	herd
16	**PatA16**	gelding	10	average	Unspecified colic	Colic pain		ConA38	partner				
17	**PatA17**	gelding	15	smal	Injury palatum	Head related pain		ConA39	partner				
18	**PatA18**	gelding	9	large	Sarcoid removal	Post operative pain		ConA46	partner				
19	**PatA19**	Jenny	26	average	Solar abcess	Orthopaedic pain		ConA43	herd	ConA44	herd	ConA45	herd
20	**PatB01**	Jenny	27	average	Solar abcess	Orthopaedic pain		ConB01	partner	ConB02	herd	ConB03	herd
21	**PatB02**	Jenny	23	average	Lameness unspecified	Orthopaedic pain		ConB04	partner	ConB05	herd	ConB06	herd
22	**PatB05**	gelding	21	average	Ulcerations mouth	Head related pain		ConB13	herd	ConB14	herd	ConB15	herd
23	**PatB06**	gelding	23	average	Solar abcess	Orthopaedic pain		ConB16	partner	ConB17	herd	ConB18	herd
24	**PatB07**	Jack	1	smal	Castration	Post operative pain		ConB19	partner	ConB20	herd	ConB21	herd
25	**PatB08**	Jack	1	smal	Castration	Post operative pain		ConB22	partner	ConB20	herd	ConB23	herd
26	**PatB09**	Jenny	14	large	Lameness unspecified	Orthopaedic pain		ConB27	partner	ConB28	herd	ConB29	herd
27	**PatB10**	gelding	22	average	Solar abcess	Orthopaedic pain		ConB24	partner	ConB25	herd	ConB26	herd
28	**PatB11**	gelding	8	average	Shoulder lameness	Orthopaedic pain		ConB30	partner	ConB31	herd	ConB32	herd
29	**PatB12**	gelding	17	average	Injured cornea	Head related pain		ConB37	herd	ConB38	herd		
30	**PatB13**	gelding	28	average	Cornea ulcer	Head related pain		ConB33	partner	ConB34	herd		
31	**PatB14**	gelding	25	average	Solar abcess	Orthopaedic pain		ConB35	partner	ConB36	herd	ConB53	herd
32	**PatB15**	gelding	31	average	Solar abcess	Orthopaedic pain		ConB40	herd	ConB52	herd		
33	**PatB16**	gelding	37	average	Laminitic / WLD^	Orthopaedic pain		ConB39	herd				

nr	Patient Donkey code	Sex	Age	Size*	General diagnose before categorisation	Disease category		name control donkey 1	type of control	name control donkey 2	type of control	name control donkey 3	type of control
34	**PatB17**	Jenny	18	average	Osteoarthritis	Orthopaedic pain		ConB41	partner	ConB42	herd		
35	**PatB19**	gelding	6	average	Laminitic / WLD^	Orthopaedic pain		ConB46	partner	ConB47	herd	ConB48	herd
36	**PatB20**	gelding	14	average	Multiple eye ulcerations	Head related pain		ConB49	partner	ConB50	herd	ConB51	herd
37	**PatB21**	gelding	28	average	Impaction colic	Colic pain		ConB54	herd	ConB55	herd		
38	**PatB22**	Jenny	17	large	Abcess coronary band	Orthopaedic pain		ConB57	partner	ConB58	herd		
39	**PatB23**	gelding	9	average	Impaction colic	Colic pain		ConB60	partner	ConB61	herd	ConB62	herd
40	**PatB24**	gelding	18	average	Impaction colic	Colic pain		ConB63	partner	ConB64	herd	ConB65	herd
41	**PatB25**	gelding	23	average	Lameness unspecified	Orthopaedic pain		ConB66	partner	ConB67	herd	ConB68	herd
42	**PatB26**	gelding	26	average	Lameness unspecified	Orthopaedic pain		ConB69	herd	ConB70	herd	ConB71	herd
43	**PatB27**	Jenny	9	average	Lameness unspecified	Orthopaedic pain		ConB72	partner	ConB73	herd	ConB74	herd
44	**PatB28**	gelding	22	average	Impaction colic	Colic pain		ConB56	herd	ConB59	herd	ConB75	herd
45	**PatC01**	Jack	8	average	Castration	Post operative pain		ConC01	herd	ConC02	herd		
46	**PatC02**	Jack	7	average	Castration	Post operative pain		ConC03	herd	ConC04	herd		
47	**PatC03**	Jack	7	average	Castration	Post operative pain		ConC05	herd	ConC06	herd		
48	**PatC04**	gelding	13	large	Cornea ulcer	Head related pain		ConC07	herd	ConC08	herd		
49	**PatC05**	gelding	8	average	Cornea ulcer	Head related pain		ConC11	partner	ConC12	herd		
50	**PatC06**	Jenny	13	average	Cornea ulcer	Head related pain		ConC09	partner	ConC10	herd		
51	**PatC07**	Jenny	18	smal	Cornea ulcer	Head related pain		ConC13	partner	ConC14	partner		
52	**PatC08**	gelding	22	average	Sarcoid removal	Post operative pain		ConC15	partner	ConC16	herd		
53	**PatC09**	gelding	10	average	Sarcoid removal	Post operative pain		ConC17	herd	ConC18	partner		
54	**PatC10**	gelding	2	average	Infection after castrationª	Post operative pain		ConC19	herd	ConC20	herd		
55	**PatC11º**	gelding	14	average	Dental extractionº	Head related pain		ConC21	partner	ConC22	herd		
56	**PatC12º**	gelding	14	average	Dental extractionº	Head related pain		ConC23	partner	ConC24	herd		
57	**PatC13**	gelding	4	average	Sarcoid removal	Post operative pain		ConC25	herd	ConC26	herd		
58	**PatC14**	gelding	14	average	Impaction colic	Colic pain		ConC27	partner	ConC28	herd		
59	**PatC15**	gelding	24	average	Colitis	Colic pain		ConC29	herd	ConC30	herd		
60	**PatC16**	Jenny	15	average	Sarcoid removal	Post operative pain		ConC31	herd	ConC32	herd		
61	**PatC17**	gelding	15	average	Dental extraction	Head related pain		ConC33	partner	ConC34	herd		
62	**PatC18**	gelding	25	average	Tympanic colic	Colic pain		ConC35	herd	ConC36	herd		
63	**PatC19**	gelding	10	average	Dental displacement	Head related pain		ConC37	herd	ConC38	herd		
64	**PatC20**	Jenny	15	average	Cornea ulcer	Head related pain		ConC39	herd	ConC40	herd		
65	**PatC21**	gelding	15	average	Lameness unspecified	Orthopaedic pain		ConC41	herd	ConC42	herd		
66	**PatC22**	gelding	21	average	Torsio	Colic pain		ConC43	partner	ConC44	herd		
67	**PatC23**	gelding	18	average	Impaction colic	Colic pain		ConC45	herd	ConC46	herd		
68	**PatC24**	gelding	15	large	Lump in neck removal	Post operative pain		ConC47	partner	ConC48	herd		
69	**PatC25**	Jenny	6	average	Sarcoid removal	Post operative pain		ConC49	partner	ConC50	herd		
70	**PatC26**	gelding	10	large	Unspecified eye problem	Head related pain		ConC51	herd	ConC52	herd		

nr	Patient Donkey code	Sex	Age	Size*	General diagnose before categorisation	Disease category		name control donkey 1	type of control	name control donkey 2	type of control	name control donkey 3	type of control
71	**PatC27**	gelding	18	smal	Cornea ulcer	Head related pain		ConC53	herd	ConC54	herd		
72	**PatC28**	gelding	3	average	Cornea ulcer	Head related pain		ConC55	partner	ConC56	herd		
73	**PatC29**	gelding	8	average	Inflamed conjunctiva	Head related pain		ConC57	herd	ConC58	herd		
74	**PatC30**	gelding	20	average	Tooth ulcer	Head related pain		ConC59	herd	ConC60	herd		
75	**PatC31**	Jenny	9	average	Sarcoid removal	Post operative pain		ConC61	partner	ConC62	herd		
76	**PatC32**	gelding	14	average	Sarcoid removal	Post operative pain		ConC63	partner	ConC64	herd		
77	**PatC33**	gelding	0	average	Injured cornea	Head related pain		ConC65	partner	ConC66	partner		
78	**PatC34**	gelding	10	average	Sarcoid removal	Post operative pain		ConC67	partner	ConC68	herd		
79	**PatC35**	Jack	7	average	Castration	Post operative pain		ConC69	partner	ConC70	herd		

*Small donkeys: <90 cm; Average donkeys: 91 - 121 cm; Large donkeys: >120 cm height at the withers
^WLD: White Line Disease
ª not included in castration dataset
º same animal admited twice with days in between for different teeth

PatB03	Deleted from data set due being allready sedated at first encounter
PatB04	Deleted from data set due to type of problem: high liver enzymes
PatB18	Deleted from data set due to type of problem: dullness

**Head related pain**	18	**Orthopaedic pain**	25	**Colic pain**	12	**Post operative pain**	24
Cornea ulceration	8	Solar abscess	10	Impaction colic	6	Sarcoid removal	10
Injured cornea	3	Laminitic -WLD	3	Torsion	2	Castration	7
Multiple eye ulcerations	2	Abscess coronary band	1	Colitis	1	Dental extractions/-displaceme	5
Mouth/teeth ulcers	1	Osteoarthritis	1	Tympanic colic	1	Removal lump	1
Palatum injury	1	Shoulder lameness	1	Unspecified	3	Infection after castration	1
Inflamed conjunctiva	1	Unspecified	9				
Unspecified	1						
